# New England Cod Collapse and the Climate

**DOI:** 10.1371/journal.pone.0158487

**Published:** 2016-07-27

**Authors:** Kyle C. Meng, Kimberly L. Oremus, Steven D. Gaines

**Affiliations:** 1 Bren School of Environmental Science and Management and Department of Economics, University of California, Santa Barbara, Santa Barbara, California, United States of America; 2 School of International and Public Affairs, Columbia University, New York, New York, United States of America; Technical University of Denmark, DENMARK

## Abstract

To improve fishery management, there is an increasing need to understand the long-term consequences of natural and anthropogenic climate variability for ecological systems. New England’s iconic cod populations have been in decline for several decades and have recently reached unprecedented lows. We find that 17% of the overall decline in Gulf of Maine cod biomass since 1980 can be attributed to positive phases of the North Atlantic Oscillation (NAO). This is a consequence of three results: i) a 1-unit increase in the NAO winter index is associated with a 17% decrease in the spring biomass of age-1 cod the following year; ii) this NAO-driven decrease persists as the affected cohort matures; iii) fishing practices appear to exacerbate NAO’s direct biological effect such that, since 1913, a 1-unit increase in the NAO index lowers subsequent cod catch for up to 19 years. The Georges Bank cod stock displays similar patterns. Because we statistically detect a delay between the NAO and subsequent declines in adult biomass, our findings imply that observed current NAO conditions can be used in stock forecasts, providing lead time for adaptive policy. More broadly, our approach can inform forecasting efforts for other fish populations strongly affected by natural and anthropogenic climatic variation.

## Introduction

Many of the world’s commercial fisheries are in decline, raising concerns about both food security [1–3] and ecosystem functioning [2, 4]. Recent literature has highlighted the need to uncover the role of environmental conditions, particularly climate variability, in driving fish populations [5–9]. It remains to be determined, however, whether such relationships can help improve stock management.

Atlantic Cod, *Gadus morhua*, one of North America’s most economically important fish stocks [10], declined precipitously starting in the 1980s [11]. In 2008, a formal stock assessment forecasted that stocks would rebound [12]; however, they were once again on the verge of collapse by 2012 [13]. In 2014, the National Oceanic and Atmospheric Administration (NOAA) instituted an unprecedented six-month ban on all Gulf of Maine stocks after the 2014 stock assessment detected historically low biomass levels [14]. Previous research has explored the role of contemporaneous environmental conditions on cod recruitment [15–22]. However, to date, such contemporaneous relationships provide little guidance on how to improve stock management, which relies on the ability to forecast future stock status.

This paper establishes that an observed climate signal, the North Atlantic Oscillation (NAO), can be used to forecast future adult cod status. This is achieved through two empirical contributions. First, using age-specific survey data for New England cod over several recent decades, we detect that NAO-driven environmental conditions have a statistically significant negative effect on cod recruitment. Second, we find that this birth-year effect persists as the cod larvae age into adulthood. This delayed effect implies that observed NAO conditions could be used to forecast future adult cod stocks. Using our statistical model, we are further able to quantify the relative contribution of the NAO to the recent collapse of these fisheries, and to provide additional evidence suggesting that fishing practices may have exacerbated the direct biological effects of the NAO.

This study is the first to detect a statistically significant effect of contemporaneous NAO conditions on cod recruitment in the Gulf of Maine and Georges Bank fisheries [17, 21, 23] which systematically persists as the cod larvae mature. Persistence of this effect is particularly important for forecasting purposes. Otherwise, if a cod cohort were able to recover from NAO’s recruitment effect as it matured (for instance, if the survival or growth rate of the affected cohort from beyond age-1 increased due to reduced competition among larval cod), the recruitment effect might dissipate over time until there were no remaining NAO effect when the cohort matures and becomes more ecologically and economically valuable. Thus, in order to establish that NAO conditions can forecast subsequent adult cod, one must (I) estimate the NAO recruitment effect from other drivers and (II) demonstrate that this effect persists over a cohort’s lifecycle.

**Fig 1:**
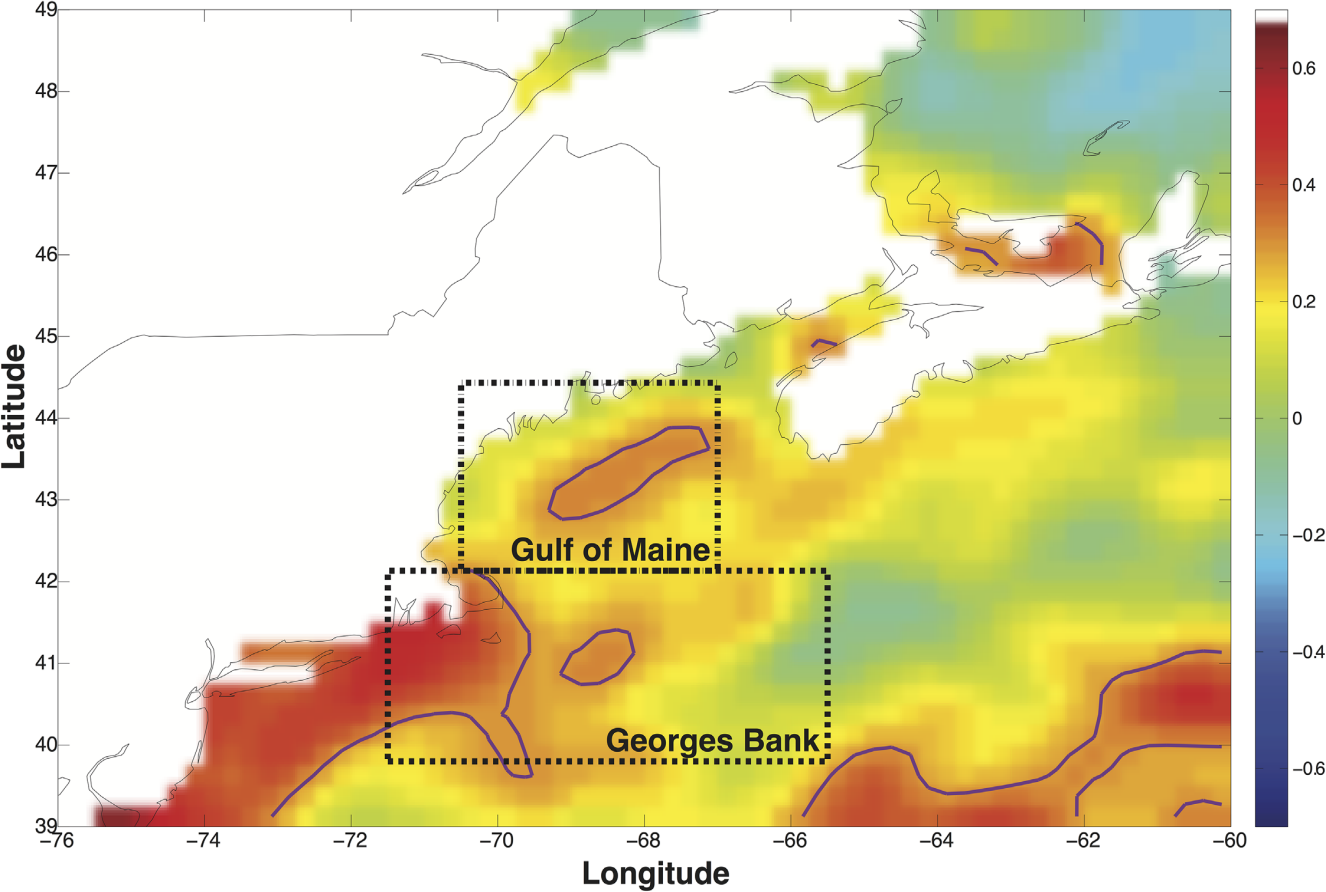
Winter NAO and Sea Surface Temperature (SST) correlation. The map shows the grid-cell-level correlation between winter (DJFM) NAO and SST from 1982 to 2013 with a quadratic time trend removed (see SI). Purple contour lines indicate areas where correlation p-value<0.1. Dashed boxes indicate the statistical area for the Gulf of Maine and Georges Bank stocks. Grid resolution is 0.25 degrees latitude x 0.25 degrees longitude. Correlation values in bar at right of map.

The North Atlantic Oscillation (NAO), defined by an index of sea-level pressure differences between the Icelandic Low and Azores High, is the dominant mode of climate variability in the North Atlantic and affects various atmospheric and oceanic processes across the region [24]. We examine the direct effects of the NAO on New England cod populations for two reasons. First, the NAO influences local environmental variables such as ocean mixing, salinity and temperature [17]. For example, a positive phase of the NAO raises sea surface temperatures (SST) off the New England coast Fig 1. These variables in turn have been documented to impact cod prey [25, 26], larval cod and cod recruitment [26, 27]. Because NAO impacts multiple local environmental variables that may simultaneously affect cod stocks, it is important to directly examine the effects of NAO fluctuations and not limit analysis to any single NAO-driven local environmental condition [5]. As supporting evidence, our analysis shows that SST, for example, contributes to a small portion of the overall NAO recruitment effect. Second, effective forecasting requires an accurately observed forecasting variable. As a hemispheric-level climatic phenomenon, the NAO index is an average of environmental conditions over a large spatial region and thus measured with less noise than any local environmental condition over a single stock [28]. Indeed, over the same sample period, we were unable to statistically detect persistent effects of birth-year SST over a cod cohort’s lifetime as we do with the NAO.

## Materials and Methods

We use time series multiple regression models based on the Ricker model [29] that combine an annual winter NAO index based on sea-level pressure (SLP) differences [24] with annual age-specific cod biomass (kg) from NOAA spring surveys of the Gulf of Maine and Georges Bank stocks [30]. Annual age-specific biomass is directly provided from the Gulf of Maine surveys and is imputed as the product of annual age-specific number of fish and annual age-specific weight from the Georges Bank surveys. First, we estimate the NAO-recruitment effect by examining the relationship between NAO conditions and the surveyed biomass of 1-year-old cod the following year. Next, to examine the persistence of these effects, we estimate the relationship between the same NAO condition and the surveyed biomass of 2-year-old cod two years later, 3-year-old cod three years later, and so on. We call this the birth-year NAO effect. If both cod stocks and NAO exhibited trending behavior during this period, our model might spuriously detect a statistical relationship between these two variables. To remove common trends, our models include a polynomial time trend to flexibly control for unobserved determinants of biomass, such as changes in fishing effort, policy, technology, and other confounding factors. Thus, to estimate unbiased birth-year NAO effects, we assume that detrended NAO variation is uncorrelated with detrended unobserved determinants of cod biomass, which is plausible given that the NAO is a naturally occurring stochastic environmental variable.

Formally, for each of the two cod fisheries, we estimate the effects of current and past NAO conditions on cod biomass (in kg) of age *a* in year t, *b*_*at*_, using the following regression model:

$$log\left(b_{at}\right)=\alpha_{a}+\sum_{\tau =0}^{L}\beta_{a\tau }NAO_{t-\tau }+\lambda 1_{at}SSB_{a,t-a}+\lambda 2_{at}log\left(SSB_{a,t-a}\right)+\sum_{p=1}^{N}\gamma_{ap}t^{p}+\epsilon_{at}$$ (1)

where *SSB*_*a*,*t*−*a*_ is spawning stock during birth year. *α*_*a*_ is a constant, *β*_*aτ*_ captures the age-specific linear effect of NAO *τ* periods ago, *λ*1 and *λ*2 capture density dependence of the recruitment effect during birth year, and *γ*_*ap*_ captures the effect of a pth-order polynomial time trend. Notice that having *log*(*SSB*_*a*,*t*−*a*_) on the right hand side of Eq 1 is a more flexible version of a standard Ricker model where the outcome variable is divided by *log*(*SSB*_*a*,*t*−*a*_), known as the survival ratio.

When *τ* = *a*, *β*_*aτ*_ captures the birth-year NAO effect, our effect of interest. (I) is established when *τ* = *a* = 1 and we estimate a statistically significant *β*_*aτ*_ indicating that NAO has a contemporaneous effect on cod recruitment. (II) is established when *τ* = *a* > 1 and we estimate a statistically significant *β*_*aτ*_ which indicates that the recruitment effect persists into adulthood. Our specification assumes that birth-year NAO has a linear effect on age-specific surveyed biomass. To ensure this is not an overly restrictive assumption, we also use a non-parametric, local polynomial regression allowing a more flexible functional form. Standard errors, *ϵ*_*at*_, use the Newey-West adjustment, which allows for serial correlation and heteroscedasticity of arbitrary form in the error terms over an optimally chosen window of time [31]. A cointegration test is performed to ensure that there are no spurious correlations due to non-stationary time-series behavior in the error term.

To quantify the contribution of the positive phase of the NAO to the observed overall decline in adult cod biomass since 1980, we first estimate an aggregate version of Eq 1 across cod ages 2 to 6, $adult\_b_{t}=\sum_{a=2}^{6}b_{at}$:

$$log\left(adult\_b_{t}\right)=\alpha_{A}+\sum_{\tau =0}^{L}\beta_{A\tau }NAO_{t-\tau }+\sum_{p=1}^{N}\gamma_{Ap}t^{p}+\epsilon_{At}$$ (2)

where *α*_*A*_ is a constant, *β*_*A*_*τ* captures the linear effect of NAO *τ* periods ago and *γ*_*Ap*_ captures the effect of a pth-order polynomial time trend. Eq 2 allows us to separate the overall decline in Gulf of Maine and Georges Bank adult cod since 1980 into the components driven by the NAO and driven by all other determinants. Specifically, our decomposition follows the procedure:

1. Estimate Eq 2 with *L* = 6 and *N* = 3 using the full sample.
2. Predict adult biomass without NAO using only secular time trends:
$\tilde{log\left(adult\_biomass_{t}\right)}=\sum_{p=1}^{N}{\hat{\gamma }}_{Ap}t^{p}$ for *t* ∈ [1980, 2013].
3. Predict adult biomass with NAO starting in 1980 and secular time trends:
$\log\hat{\left(adult\_biomass_{t}\right)}={\hat{\alpha }}_{A}+\sum_{\tau =0}^{L}{\hat{\beta }}_{A\tau }NAO_{t-\tau }+\sum_{p=1}^{N}{\hat{\gamma }}_{Ap}t^{p}$ for *t* ∈ [1980, 2013].

Observe that while Eq 2 estimates ${\hat{\beta }}_{A\tau }$ using detrended NAO variation, step 3 predicts adult biomass by multiplying ${\hat{\beta }}_{A\tau }$ with observed NAO. This allows us to quantify the effect of all observed NAO variation. To get the percentage contribution in the overall adult biomass decline due to the NAO from 1980 to 2010, we calculate the following:

$$\text{NAO}\;\text{contribution}=\frac{\left({\hat{adult\_b}}_{2010}-{\hat{adult\_b}}_{1980}\right)-\left({\tilde{adult\_b}}_{2010}-{\tilde{adult\_b}}_{1980}\right)}{\left(adult\_b_{2010}-adult\_b_{1980}\right)}$$ (3)

In practice, due to noisy biomass values, we take the average values over the first 3 and last 3 years of the sample period when applying Eq 3. Results for cod catch follow the same approach but with log catch as the outcome variable and with lagged NAO regressors for up to 20 years.

## Results

**Fig 2:**
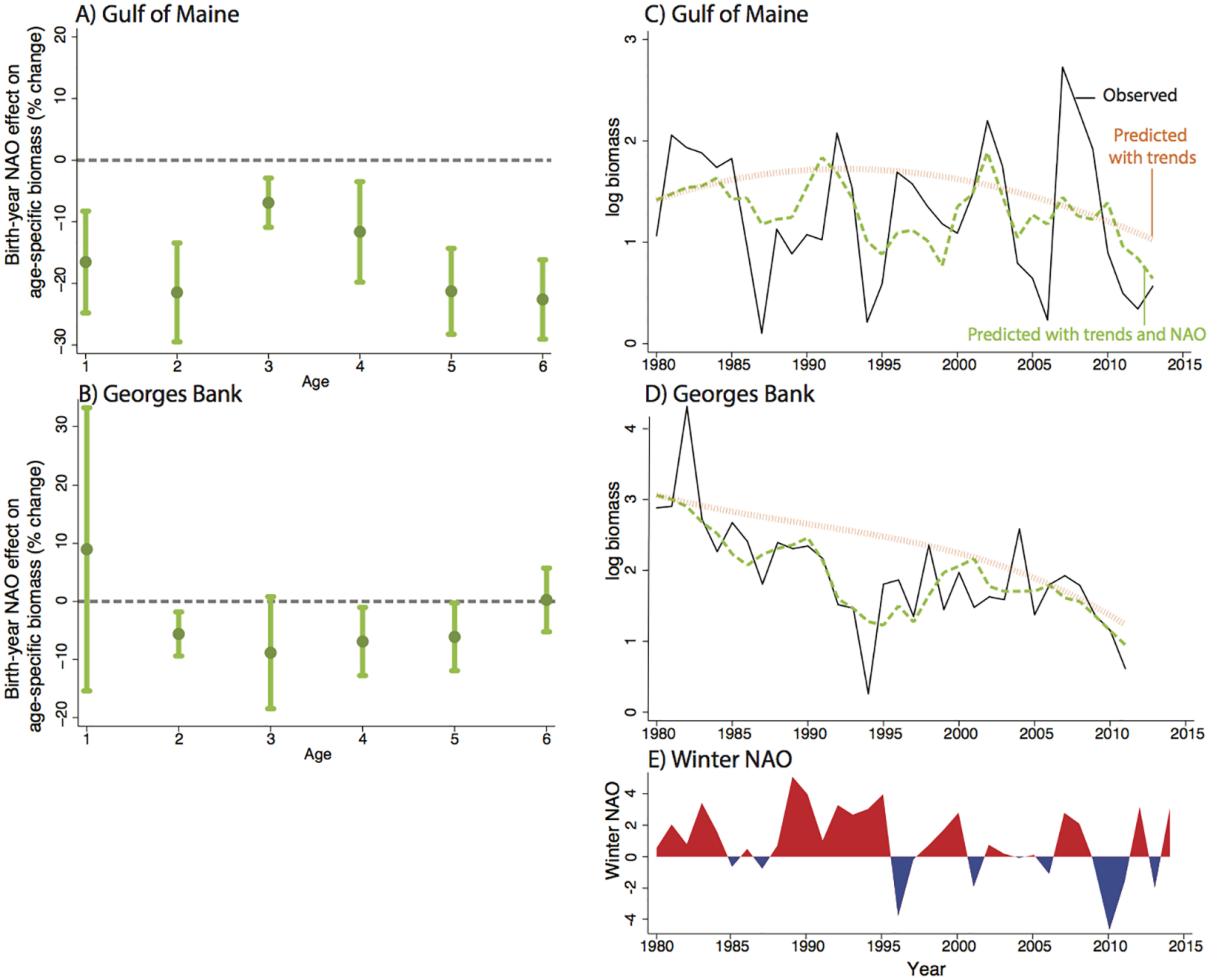
Birth-year NAO effect on cod biomass. Panels **(A)** and **(B)** show regression coefficients representing the effect of a 1-unit increase in birth-year NAO on a cod cohort as it matures from age 1 to 6 for the Gulf of Maine and Georges Bank stocks, respectively. Each coefficient comes from a separate multiple regression model (see Eq 1). 90% confidence interval shown. Panels **(C)** and **(D)** show observed surveyed log adult biomass (ages 2–6) (black line), predicted log adult biomass using only secular time trends (orange line), and predicted log adult biomass using both secular time trends and observed NAO (green line). Decomposition follows Eqs 2 and 3. Panel **(E)** shows observed NAO variation.

Panel (A) of Fig 2 plots the regression coefficients *βατ* when *τ* = *α* from Eq 1, or the birth-year NAO effect, estimated separately for cod ages 1 to 6. There is a negative relationship between the NAO index and cod biomass. For the Gulf of Maine stock from 1971–2013, a 1-unit increase in the NAO index, which increases winter SST by .045°C (Table A in S1 File), during a cohort’s birth year is associated with a 17% drop in surveyed biomass for that cohort at age 1 (results are similar when modeling the ratio of age-1 biomass over spawning stock, or survival ratio). Going from left to right of Panel (A) of Fig 2, we see that this effect persists as the cohort matures to age 6, with statistically significant effects ranging from a 7% to 23% decrease in biomass (Table C in S1 File). A similar pattern of results is shown for the Georges Bank stock from 1979–2011 in Panel (B) of Fig 2 (Tables B and G in S1 File), though results are noisier for data limitation reasons detailed in the Supplemental Information (S1 File). Birth-year NAO effects are unaffected by replacing the SLP-based winter NAO index with a principal component-based winter NAO index (Fig A in S1 File).

To show why it is important to directly model the effects of the NAO rather than that of a local environmental condition, we re-estimate Eq 1 replacing the NAO terms with local average winter (DJFM) sea surface temperatures (SST) over each stock. For the Gulf of Maine, we do not systematically detect a birth-year SST effect across all ages, finding effects only for age 2–4 cod (Table P in S1 File). For age 2 cod, this implies that the partial birth-year NAO effect through SST is one-quarter the total NAO effect. Specifically, (−1.1 * .045)/ − .22 = .23, where -0.22 is our estimated NAO effect from Column (3) of Table C in S1 File, -1.1 is our estimated SST effect from Column (3) of Table P in S1 File, and 0.045 is the linear relationship between winter NAO and SST from Column (3) of Table A in S1 File. This suggests that NAO is affecting cod populations through other environmental variables in addition to changes in SST. We do not detect a birth-year SST effect on biomass for any age in Georges Bank (Table Q in S1 File).

Our birth-year NAO effects are robust to assumptions over the functional form of NAO effects (Fig B in S1 File), the order of the polynomial time trends (Tables D and H in S1 File), and the number of included lagged NAO terms (Tables E and I in S1 File). They are also robust to controlling for past catch (Tables C and G in S1 File, see further explanation in S1 File). For both fisheries, NAO appears primarily to have a birth-year effect. We detect some contemporaneous effects of NAO on adult cod, but they do not persist consistently over time (Tables C and G in S1 File). Furthermore, we find weak, though inconclusive, evidence that NAO during the birth-year of one generation lowers the biomass of subsequent generations (Tables F and J in S1 File). Previous papers have argued that the relationship between environmental conditions and recruitment may be changing over time [17]. We do not find that the NAO-recruitment relationship is trending over time (Fig C in S1 File) for the Gulf of Maine stock, the stock with the longer time series, though the relationship may exhibit decadal-scale cyclicality. Finally, we do not find evidence of an NAO-recruitment effect on fall-spawning cod larvae, which is known to be a different population than spring-spawning cod (Tables R and S in S1 File) [26, 32].

**Fig 3:**
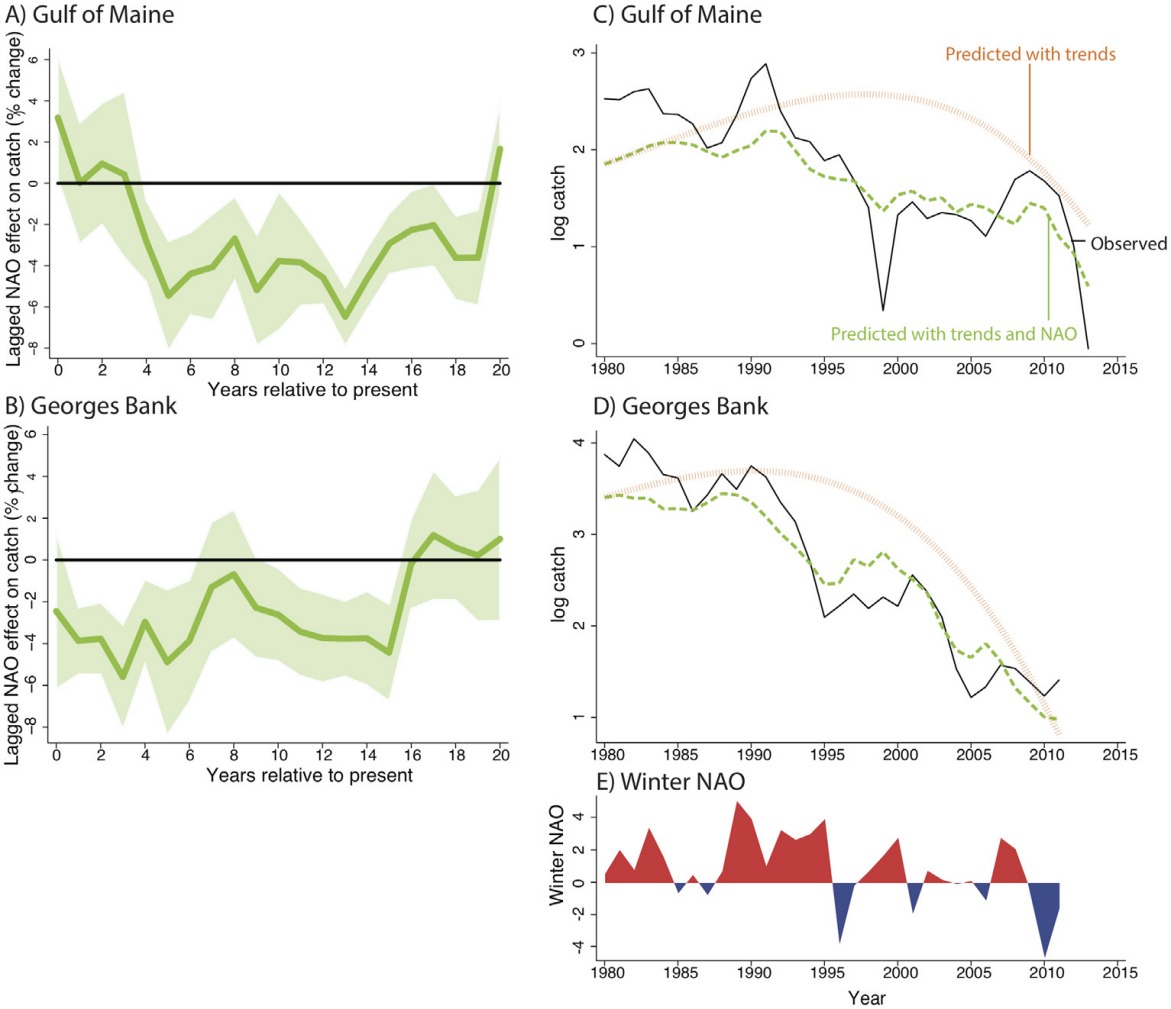
Effects of current and past NAO on cod catch. Panels **(A)** and **(B)** show regression coefficients from a multiple regression model of commercial cod catch on current and past NAO variability for the Gulf of Maine and Georges Bank stocks, respectively. The x-axis represents the number of years prior that a 1-year increase in NAO occurred. 90% confidence interval shown. Panels **(C)** and **(D)** show observed log catch (black line), predicted log catch using only secular time trends (orange line), and predicted log catch using both secular time trends and observed NAO (green line). Panel **(E)** shows observed NAO variation.

NAO was in a repeated positive phase from 1980–1995 (Fig 2, Panel (E)) and the index has trended more positive overall from the mid-1970s to today. Though consensus has not been reached on why NAO has been trending upward, some predict an increased frequency of positive NAO conditions under increasing greenhouse gas emissions over the next century [33, 34]. We explore the contribution of the positive phase of the NAO from 1980–2013 to the overall decline in adult biomass by estimating Eq 2 and applying our decomposition method from Eq 3, which separates predicted adult biomass with and without NAO effects during this period. The black line in Panels (C) and (D) of Fig 3 shows observed adult biomass for the two fisheries respectively. The orange line represents a “counterfactual” biomass trajectory with the influence of NAO removed and thus driven only by trends in latent factors, including changes in spawning biomass and fishing effort, that are unrelated to the NAO. The green line represents biomass predicted by both trends and observed NAO such that the difference between the green and orange lines represents the isolated contribution of the NAO to biomass. Using Eq 3, we find that the NAO has contributed 17% and 9% of the overall decline in adult biomass in the Gulf of Maine and Georges Bank stocks since 1980, respectively (see S1 File, Tables K and L in S1 File). The green line (in Panel C and D) depicts how the inter-annual variation in NAO conditions (Panel E) drives much of the inter-annual variation in adult cod biomass.

While we are able to isolate the birth-year NAO effect from other drivers of adult cod biomass, including fishing effort unrelated to NAO, it is more difficult to distinguish between the direct biological effects of birth-year NAO and any indirect effects of birth-year NAO mediated through fishing effort. For example, it is conceivable that NAO could indirectly affect cod biomass via local environmental conditions that affect fishing effort. Our statistical model is unable to isolate such indirect effects though they could potentially lessen, maintain, or amplify the direct biological effect of birth-year NAO. For the birth-year affected cohort, we do not find evidence of this additional indirect NAO impact. First, we observe that the birth-year NAO effect is of relatively similar magnitudes for cohorts ages 1 to 6, suggesting that fishing effort in response to the NAO as the cohort matures is not changing the initial birth-year effect. Second, directly controlling for past catch does not alter birth-year NAO effects at each age (Tables C and G in S1 File).

To examine whether NAO effects are amplified or mitigated by fishing practices, we turn to data on commercial cod catch, which is a function of both cod biomass summed across adult cohorts and fishing effort. This analysis provides a key benefit: The New England cod fisheries have one of the longest catch time series in the world: over 100 years of data, covering the entire 20th century and providing a sample period that allows for detection of very long-run effects. Panels (A) and (B) of Fig 3 plot the coefficients from a single regression of commercial catch on current and past NAO using data spanning the period 1913 to 2013 for the Gulf of Maine stock and 1913 to 2011 for the Georges Bank stock, respectively (see S1 File). In the Gulf of Maine stock, we find that a 1-unit increase in the NAO index during this period drives a 3% to 6% decline in catch that lasts up to 19 years (Table M and N in S1 File). We find persistent effects of similar magnitude for up to 15 years after a 1-unit increase in NAO for the Georges Bank stock (Table M and O in S1 File). Using the same decomposition method shown in Panels (C) and (D) of Fig 2, Panels (C) and (D) of Fig 3 indicate that the positive phases of the NAO since 1980 have contributed 32% and 7% of the overall decline in catch in the Gulf of Maine and Georges Bank stocks, respectively.

## Discussion

There are two possible explanations for the long persistence of past NAO conditions on cod catch. First, this persistence may be driven entirely by biological dynamics if birth-year NAO lowers adult spawning of one generation and thus recruitment for the next generation. However, as already noted, we find weak evidence of intergenerational effects. Alternatively, it is possible that fishing effort has historically reacted inadvertently to birth-year NAO-driven drops in a particular cohort by increasing fishing effort uniformly across all adult cohorts. Such a practice would induce the spillover of birth-year NAO effects of a particular cohort onto younger and older cod and extend the legacy of past NAO variability on commercial catch. As such, this evidence supports an emerging literature noting that fish stocks may be affected by an interaction of the direct biological effect of environmental drivers and the indirect, possibly unintended, effect of fishing effort in response to these drivers [22, 35].

This paper is unable to isolate the specific biological mechanism through which NAO fluctuations affect cod recruitment and subsequent cod biomass. While this is basis for future work, simply establishing the birth-year NAO effect has an important fishery management implication. Because the birth-year NAO effect persists as a cohort matures, one can use an observed NAO index to forecast future adult cod biomass without needing to forecast the NAO itself or fully understand the precise biological pathway. One simple way to incorporate our finding is to adjust the recruitment parameter in stock assessment models to reflect the current observed state of the NAO. Incorporating NAO’s forecasting ability into stock assessment models may be particularly timely given the positive-phase NAO in 2012, 2014 and 2015 of 3.17, 3.10 and 3.56 *σ*, respectively.

More broadly, the forecasting potential described in this paper may be relevant to other fisheries in which climatic conditions affect fish larvae in ways that persist over many years to impact future adult fish populations. Many ecological studies of recruitment in unfished species have shown strong connections between successful recruitment of larvae and such observable environmental cycles [36, 37]. Our analysis may also serve as an analogue for understanding the future impacts of anthropogenic climate change, which is projected both to increase average SST off the New England coast and to alter the frequency and magnitude of climatic variation in other parts of the globe.

## Supporting Information

S1 File Appendix containing all additional tables and figures. (PDF)
