# Supplementary material for: New England Cod Collapse and the Climate
Source: PLoS One. 2016 Jul 27;11(7):e0158487. doi: 10.1371/journal.pone.0158487 (PMC4963117; doi:10.1371/journal.pone.0158487)
Supplement: S1 File — (PDF) [file pone.0158487.s001.pdf]

Supplemental Information for

New England Cod Collapse and the Climate

Kyle C. Meng[*], Kimberly L. Oremus[†], and Steven D. Gaines[‡]

June 2016

This Supplementary Information appendix contains the following four sections:

S.1 Data sources

S.2 Statistical models

S.3 Supplemental figures

S.4 Supplemental tables

This Supplemental Information section discusses data sources, statistical methods, and robustness tests that are referred to in the main text. Replication files for all results can be found at Dryad data repository with DOI number: doi:10.5061/dryad.k86v1

S.1 Data sources

S.1.1 NAO and SST data

We primarily use the Hurrell winter (DJFM) station-based index of the North Atlantic Oscillation (NAO) which is based on the difference of normalized sea level pressure between Lisbon, Portugal and Reykjavik,

[*]Corresponding author, Bren School of Environmental Science and Management and Department of Economics, University of California, Santa Barbara, Santa Barbara, California 93106, USA.

[†]School of International and Public Affairs, Columbia University, New York, New York, 10027, USA

[‡]Bren School of Environmental Science and Management, University of California, Santa Barbara, Santa Barbara, California 93106, USA.

Iceland.[1] This index was obtained annually from 1864-2013.[i] As a validation check, we also collected a principle component-based DJFM NAO index for the same time period.[ii]

To examine the relationship between winter NAO and winter (DJFM) sea surface temperatures (SST) over the Gulf of Maine and Georges Bank fisheries, we use the NOAA OI SST V2 High Resolution Dataset which provides daily SST values in degrees Celsius over a 0.25 degree latitude by 0.25 degree longitude grid from 1/1/1981 to the present.[2] For a given year, we construct an annual winter SST measure by averaging daily SST observations from December of the previous year to March of the given year over each grid cell. After removing time trends (see Section S.2.1), we then correlate grid-cell-level winter SST with winter NAO for the period 1982-2013 to produce Figure 1 in the main text.

S.1.2 Cod surveyed biomass and catch

Surveyed age-specific biomass during the spring for the Gulf of Maine and Georges Bank Atlantic cod fisheries come from the latest stock assessments, primarily the 55th Northeast Regional Stock Assessment Workshop (SAW) produced by the Northeast Fisheries Science Center (NEFSC).[3,4] We use data directly reported from stock surveys which may be subject to various biases related to survey design.[iii] For the Gulf of Maine, annual spring biomass surveys were conducted for each age cohort from 1970-2012 (from Table A58 in NEFSC (2013)) and extended to 2013 (using Tables 1.22 and 1.26 in NEFSC (2014)). Unfortunately, age-specific total biomass is not available from spring surveys of the Georges Bank. Instead, we obtain the number of fish collected by cohort (also known as abundance) from the annual spring surveys (Table B15 in NEFSC (2013)) and multiply each value by the yearly-average weight by age from the annual spring surveys (Table B17a in NEFSC (2013)) to impute age-specific biomass from 1978-2011. Fall survey data for both stocks was pulled from the same sources: Table A59 for Gulf of Maine and Table A60 for Georges Bank.

We restrict attention to only cod ages 1 to 6 and avoid modeling NAO effects on older cod because they are sampled less frequently in stock assessment surveys. Over our respective sample periods, 8.1% of all cod surveyed are age 7 or older in the Gulf of Maine. For Georges Bank, that percentage is 5.2%. For cod age cohort 1 to 6, biomass values (in kg) for each fishery and year are almost all strictly positive.[iv]

Total commercial catch (also known as landings) from U.S. and foreign boats was also obtained from a combination of NEFSC stock assessment reports. Because the 55th SAW only reported commercial catch

[i] Available here: https://climatedataguide.ucar.edu/sites/default/files/climate_index_files/nao_station_djfm_0.txt

[ii] Available here: https://climatedataguide.ucar.edu/climate-data/hurrell-north-atlantic-oscillation-nao-index-pc-based

[iii] Available here: http://nefsc.noaa.gov/publications/crd/crd1311/

[iv] The only exception are Gulf of Maine age-1 biomass in 2011 and age-6 biomass in 1987 which are recorded as zero values. Given the positive values in years prior and after these zero values, we believe this is due to recording error. To avoid missing values when we apply a log-transformation to biomass, we replace these two zero values with an imputed value based on a linear interpolation of age specific biomass from the previous and following data years. This minor data imputation is not essential to our results.

starting in 1932 for the Gulf of Maine (Tables A8-A9 in NEFSC (2013)), we augment our data to include an earlier[5] and the latest stock assessment[4] yielding a continuous catch time series for 1893-2013. Similarly, commercial landings for the Georges Bank fishery (Table B1 in NEFSC (2013)) was extended back to 1893 using an earlier stock assessment[6] to obtain a continuous catch time series for 1893-2011.

S.2 Statistical models

This section describes the statistical models used to establish the following empirical relationships for the Gulf of Maine and Georges Bank cod fisheries: 1) the effect of winter NAO on winter SST, 2) the effect of past and current winter NAO on age-specific surveyed biomass, 3) the effect of past and current winter NAO on surveyed adult biomass (summed over ages 2-6), and 4) the effect of past and current winter NAO on commercial catch. Each model is also presented with related diagnostic checks. For all our results, we use a distributed lag time-series linear regression model.

S.2.1 Modeling NAO effects on SST

To establish the relationship between winter NAO and winter SST, we first obtain an average annual winter SST value, SST_t, for each fishery by averaging grid-cell-level SST values from the NOAA OI SST Dataset within the spatial bounds of each fishery as defined by the NEFSC (see fig. 1 in main text). We run the following regression model:

$$SST_t = \omega + \phi NAO_t + \sum_{p=1}^{N} \mu_p t^p + \epsilon_t \tag{S.1}$$

where ω is a constant, ϕ captures the linear effect of current winter NAO and μ_p captures the effect of a pth-order polynomial time trend. Standard errors use the Newey-West adjustment which allows for serial correlation and heteroscedasticity of arbitrary form in the error terms over an optimally chosen window of time.[7,8] We estimate Equation S.1 separately for each fishery during the 1982-2013 period, which covers the years with available high resolution SST data. Tables A and B show estimates of ϕ and related statistics of Equation S.1 for the Gulf of Maine and Georges Bank fisheries respectively. SST during this sample period exhibited trending behavior and thus needed to be detrended. To determine the polynomial order of the time trend, N, we use the Akaike Information Criteria (AIC),[9] which when minimized captures a model's overall goodness of fit while penalizing additional terms with limited explanatory power. For both fisheries, we observe that the AIC statistic drops when a time trend of second-order or higher is included in Equation S.1. Importantly, we detect a strong positive relationship between winter NAO and winter SST. The results

in Tables A and B correspond to the spatially explicit correlation map shown in Figure 1 in the main text and provides justification for the joint detrending of NAO and SST values using a quadratic time trend.

S.2.2 Modeling NAO effects on age-specific surveyed biomass

In order for current NAO events to forecast subsequent adult cod biomass, we must establish 1) that NAO lowers the survival rate of cod larvae and 2) that this birth-year NAO effect persists as the cod cohort matures. Testing for the persistent of birth-year NAO impacts as a cohort matures helps to rule out possible mean-reverting patterns due to higher growth rates at lower stock levels. For each of the two cod fisheries, we estimate the effects of current and past NAO events on cod stock (in kg) of age a in year t, $biomass_{at}$ by running the following Ricker time-series regression:

$$log(b_{at}) = \alpha_a + \sum_{\tau=0}^{L} \beta_{a\tau} NAO_{t-\tau} + \lambda 1_{at} SSB_{a,t-a} + \lambda 2_{at} log(SSB_{a,t-a}) + \sum_{p=1}^{N} \gamma_{ap} t^p + \epsilon_{at} \qquad \text{(S.2)}$$

where $SSB_{a,t-a}$ is spawning stock during birth year. α_a is a constant, $\beta_{a\tau}$ captures the age-specific linear effect of NAO τ periods ago, $\lambda 1$ and $\lambda 2$ capture density dependence of the recruitment effect during birth year, and γ_{ap} captures the effect of a pth-order polynomial time trend. There are three classes of NAO effects. When $\tau = a$, $\beta_{a\tau}$ captures the effect of an earlier NAO event that occurred during a cohort's birth year. We call this the birth-year NAO effect and is our primary effect of interest. When $\tau < a$, $\beta_{a\tau}$ captures the effect of NAO on cod that is age-1 and older. We call this the post-birth-year or adult NAO effect. Finally, when $\tau > a$, $\beta_{a\tau}$ captures the effect of NAO on the biomass of subsequent generations due to a drop in the spawning stock biomass. We call this the pre-birth-year or intergenerational NAO effect. As we will show, we find the most consistent evidence for a birth-year NAO effect across both cod fisheries. Standard errors use the Newey-West adjustment which allows for serial correlation and heteroscedasticity of arbitrary form in the error terms over an optimally chosen window of time.[7,8]

In our preferred models, we include current and lagged NAO terms up to and including birth-year NAO such that L must be no smaller than the age of the cohort, a. We do this for two reasons. First, it may be that age-specific biomass exhibits serial correlation. Because NAO is an autoregressive oscillation, this implies that excluding past NAO events may result in omitted variables bias. Second, including NAO terms after a cohort's birth year allows us to examine whether there are systematic post-birth-year NAO effects.

Tables C and G show estimates for Equation S.2 for each age-cohort for the Gulf of Maine and Georges Bank fisheries respectively. We restrict the sample period to be constant across cohort models within a fishery. Our coefficient of interest is the birth-year NAO effect on age-specific biomass which is shaded in gray. For age-1 cod, that effect is shown by the coefficient on NAO_{t-1}. Likewise for age-2 cod, that effect is

captured by the coefficient on NAO_{t-2} and so on. The coefficients in bold are from our preferred statistical model and are plotted in Panels (A) and (B) of Figure 2 in the main text. Each model includes a 3rd-order polynomial time trend and the same number of lagged NAO terms as the cohort's age. In the next subsection, we justify these and other modeling decisions.

For the Gulf of Maine fishery, a 1-unit increase in the NAO index during a cohort's birth year is associated with a -13% change in surveyed biomass for that cohort at age 1. This effect persists as the cohort matures to age 6, with statistically significant effects ranging from -8 to -19% (Table C). Because biomass is imputed and not directly observed for the Georges Bank fishery, birth-year NAO effects are noisier for age-1 cod. However, we find that a 1-unit increase in birth-year NAO similarly lowers the surveyed biomass of cod ages 2 to 5 by -9 to -16% (Table G). This persistent effect appears to dissipate by age 6, though an effect of -17% is detected for NAO occurring five years ago which may capture the birth-year NAO effect. This may be due to errors in age assignment during cod surveys as the age of older fish may be harder to determine. For both fisheries we pick up some post-birth-year NAO effects but they do not persist systematically like birth-year NAO effects.

We preform the same analysis on fall survey cod biomass data. There is a genetically different population that spawns in the fall than in the spring. For GOM, significant effects are seen for age-2 cod and above, but not for age-1 cod (Table R). This is consistent with the fact that NAO is a mode of winter climate variability, so it should theoretically only impact spring recruitment. Results for Georges Bank stock do not show significant NAO-birth-year effects for any age cohort (Table S)

We also estimate Eq. S.2 using an alternative principal component-based (PC) DJFM NAO index which allows for spatial shifts in the pressure centers of the NAO. This is for comparison with the benchmark station-based Hurrell index, which uses sea-level pressure (SLP) differences defined over fixed spatial areas. Figure A replicates Panels (A) and (B) of Figure 2 in the main text showing the birth-year NAO effect for Gulf of Maine (top panel) and Georges Bank (bottom panel) stocks, respectively. To make the NAO indices comparable, both indices are standardized to zero mean and unit variance prior to estimating Eq. S.2. Estimates using the SLP-based NAO index are in green while those using the PC-based index are in blue. Birth-year NAO effects do not systematically differ according to which NAO index is used.

Model selection tests

Order of polynomial time trends: We must determine N in Equation S.2, the order of the polynomial time trend for each age cohort. If both cod stocks and NAO exhibited trending behavior during this period, our model might detect a statistical relationship between these two variables that is driven by a common trend. Results in Tables C and G address this issue by jointly removing a 3rd-order polynomial time trend.

In Tables D and H, we examine whether higher or lower order polynomial trends affect the stability of the birth-year NAO effect for each age cohort and fishery separately. Specifically, we vary the order of included time-trend terms from 1 to 5 across Columns (1)-(5) respectively. Each horizontal panel shows a different age cohort; thus each "cell" presents the birth-year NAO effect and related statistics from separate regressions.

Table D demonstrates the birth-year NAO effect is relatively stable regardless of the order of the polynomial time trend for each age cohort for the Gulf of Maine fishery with the coefficients in bold corresponding to that shown in Panels (A) and (B) of Figure 2 in the main text. Furthermore, the Akaike Information Criteria[9] is similar in magnitude across the columns. Table D also summarizes results from a Dickey-Fuller Generalized Least Square (DF-GLS)[10] which tests whether our time-series model exhibits unit-root behavior. The presence of a unit root means the time series variable may not be stationary and can lead to spurious correlations.[11] The DF-GLS test examines a model's estimated residual against the null hypothesis that there is a unit root.[v] With the exception of age-4 and age-5 cohort models, we reject the presence of a unit root for most other age-cohort models.

For the Georges Bank fishery, age 2 to age 5 birth-year NAO effects are also relatively stable across trend specifications (Table H). However, there appears to be a unit root for age 2, age 3, and age 6 cohorts models. We posit that a unit root may have been artificially generated due to the imputed nature of Georges Bank surveyed biomass discussed in Section S.1.2.

Number of lagged NAO terms and intergenerational effects: Equation S.2 requires choosing L, the number of NAO lag terms. Our baseline specification sets $L = a$, that is it includes post-birth-year NAO terms but excludes pre-birth-year NAO terms. Table E examines whether the birth-year NAO effect is sensitive to alternative lag NAO structures for the Gulf of Maine fishery by estimating models that exclude post-birth-year NAO terms and jointly include both post-birth-year and pre-birth-year NAO terms. The presentation structure is similar to that of Table D. The first column estimates a model with only a birth-year NAO term and excludes post-birth-year NAO terms. Each subsequent column includes all post-birth-year NAO terms as well as in additional pre-birth-year NAO term. For simplicity of presentation, all models include a 3rd-order polynomial time trend. The pattern of results are mostly similar for other trend specifications (not shown). Again, the coefficients in bold correspond to that shown in Panels (A) and (B) of Figure 2 in the main text. The birth-year NAO effect appears stable regardless of the exclusion of post-birth-year NAO terms and the inclusion of pre-birth-year NAO terms.

Table E only shows the birth-year NAO effect when additional pre-birth-year NAO events are included. In Table F, we display the additional pre-birth-year effects to explore if NAO has any intergenerational

[v]The optimal lag length chosen for each DF-GLS tests is based on a AIC statistic.

effects for the Gulf of Maine. If an NAO event reduces a birth-year cohort's biomass and this reduction persists to when the cohort is reproductively mature, then the biomass of that cohort's offspring may also be negatively affected. This implies that birth-year NAO effects may transmit past a single generation. Atlantic cod typically reach reproductive maturity beginning at age 2.[6] If intergenerational effects exist, we may detect the adverse impacts of NAO two years prior to the birth of a particular cohort. It is worth noting, however, that intergenerational effects may not necessarily follow a clear 2-year interval as reproduction occurs continuously once a fish reaches reproductive maturity. Thus, our tests for intergenerational effects are likely to be imprecise.

In Table F, we extend the number of lags for all age cohorts to age 4, displaying all NAO coefficients. We do not estimate further lags given our limited sample size and so are unable to detect intergenerational effects for cohorts older than age 4. Table F provides some, though weak, evidence that birth-year NAO effects persist beyond a single generation for the Gulf of Maine. In Column (1) we find that age-1 biomass decreases in response to NAO events four and six years prior, roughly corresponding to NAO events felt by one and two earlier generations. We also find a one-generation effect for age-2 and age-4 cod, but fail to find an intergenerational effect for age-3 cod. For the Georges Bank fishery, we also find that the birth-year NAO effect is stable to the exclusion of post-birth-year NAO terms and the inclusion of pre-birth-year NAO effects (Table I). We also find even weaker evidence of intergenerational effects with a one-generation effect detected for age-3 and age-4 cod only (Table J).

Nonlinearity: Equation S.2 implicitly assumes that birth-year NAO has a linear effect on age-specific surveyed biomass. We test for whether linearity is an overly restrictive assumption in Figure B for both fisheries. Following Equation S.2, we first regress $log(biomass_{at})$ on a constant, all post-birth-year NAO terms, birth-year spawning stock in levels and log, and a 3rd-order polynomial time trend and obtain the residuals. We perform the same partialling-out procedure for $NAO_{t-\tau}$. We then fit the two residuals using a bivariate local polynomial regression allowing for data-driven flexible functional forms.[12] Panel (A) of Figure B shows the bivariate relationship between surveyed biomass for ages 1-6 cod and birth-year NAO for the Gulf of Maine. For the Gulf of Maine, the partialed-out age-specific biomass has an approximately linear relationship with partialed-out birth-year NAO for all age cohorts. For Georges Bank, Panel (B) of Figure B shows similar linearity for the relationship between age-specific biomass and birth-year NAO effects with the exception of age 1 and age 3 biomass.

Time-varying effects Equation S.2 implicitly assumes that the birth-year NAO effect is constant over the course of the sample period. Previous papers have noted that environmental-recruitment relationships

may be changing over time.[13] We statistically test for time-varying effects by conducting a rolling-window analysis of our birth-year NAO recruitment effect (i.e. on age-1 cod). Figure C plots coefficients from 20-year wide estimation windows for the Gulf of Maine cod, the stock with the longer time-series data. There is no linear trend, positive or negative, in the relationship over time during the past four decades. There does appear to be non-trending, low-frequency cyclicality in the relationship whose mean is captured by my full sample estimates. Figure C shows my full sample confidence intervals as red lines.

Controlling for previous year catch and adult biomass: One frequently modeled determinant of biomass is past catch. The models shown in Columns (2), (5), (7), (9), (11), and (13) of Tables C and G include an additional term for previous year's catch to Equation S.2 for each fishery. Our birth-year NAO effects are largely unchanged after controlling for previous year's catch.

However, our preferred specification for Equation S.2 and our results shown in Figure 2 explicitly omits terms for past catch. We omit catch because as Table M demonstrates, past NAO events lowers catch through a combination of direct effects on adult biomass and indirect effects on fishing effort. Controlling for past catch in Equation S.2 thus leads to a "proxy control" problem (see p. 66 of[14]) and may result in biased estimates of birth-year NAO effects. This problem becomes more pronounced as longer lags of past catch are included in Equation S.2 given the strong persistence of past NAO events on catch shown in Table M.

To demonstrate that the birth-year NAO effect on cod recruitment is not being confounded by adult cod biomass, we augment our model of age-1 surveyed biomass to include the previous year's surveyed adult biomass (summed over ages 2-6) in Column (3) of Tables C and G.[vi] Again, our birth-year NAO effect is largely unaffected.

S.2.3 Modeling winter SST effects on age-specific spring-surveyed biomass

To examine the effects of local winter SST on age-specific cod biomass, we estimate a variant of Eq. S.2 replacing all NAO terms with SST terms. Birth-year winter SST effects were not detected for year-1 cod and do not show persistence after year-4 (Table P). We do not detect a birth-year SST effect for any age in Georges Bank (Table Q).

[vi] We prefer to use surveyed adult biomass as a proxy for "spawning stock biomass" (SSB) because constructing SSB requires cohort-specific weights that are typically based on modeling assumptions.

S.2.4 Decomposing NAO's effect on adult cod biomass decline since 1980

NAO has generally been in a positive phase over the last few decades (Figure 2, Panel (E)). We are interested in examining the contribution of these recent positive NAO events on the observed overall decline in adult cod biomass since 1980 as shown in Panels (C) and (D) of Figure 2. We first estimate an aggregate version of Eq. S.2 across cod ages 2-6, $adult\_b_t = \sum_{a=2}^{6} b_{at}$:

$$log(adult\_b_t) = \alpha_A + \sum_{\tau=0}^{L} \beta_{A\tau} NAO_{t-\tau} + \sum_{p=1}^{N} \gamma_{Ap} t^p + \epsilon_{At} \tag{S.3}$$

where α_A is a constant, $\beta_A\tau$ captures the linear effect of NAO τ periods ago and γ_{Ap} captures the effect of a pth-order polynomial time trend. Following our earlier age-specific biomass regressions, we include up to 6 lagged NAO terms such that $L = 6$. Odd numbered columns in Table K show estimates of β_τ for the Gulf of Maine fishery. Results are equivalent to a biomass weighted sum of estimates from Table C. Using a 3rd-order polynomial time trend displayed in Column (5), a 1% increase in NAO 4, 5, and 6 years ago lowers current adult cod biomass by 8%, 6% and 11% respectively. These effects are relatively stable across 2nd-4th order polynomial time trend specifications but do change when only a linear time trend is included. Similar results are shown for the Georges Bank fishery in Table L. With a 3rd-order polynomial time trend as shown in Column (5), a 1% increase in NAO 3, 4, 5, and 6 years ago lowers current adult cod biomass by 8%, 10%, 9% and 7% respectively. Results are largely robust to the order of the polynomial time trend. Even numbered columns of Tables K and L also include an additional control for previous year catch. As discussed above, this specification contains a "proxy control" problem and is not preferred. For both fisheries, our results are unaffected by the inclusion of previous year catch.

To construct the decomposition shown in Panels (C) and (D) of Figure 2, we perform the following procedure:[15]

1. Estimate Eq. S.3 with $L = 6$ and $N = 3$ using the full sample.

2. Predict adult biomass without NAO using only secular time trends:
$log(\widetilde{adult\_b_t}) = \sum_{p=1}^{N} \hat{\gamma}_{Ap} t^p$ for $t \in [1980, 2013]$.

3. Predict adult biomass with NAO starting in 1980 and secular time trends:
$log(\widehat{adult\_b_t}) = \hat{\alpha}_A + \sum_{\tau=0}^{L} \hat{\beta}_{A\tau} NAO_{t-\tau} + \sum_{p=1}^{N} \hat{\gamma}_{Ap} t^p$ for $t \in [1980, 2013]$.

Panels (C) and (D) of Figure 2 plot the observed adult biomass, $log(adult\_b_t)$ (black line), the predicted adult biomass using only the secular time trend, $log(\widetilde{adult\_b_t})$ (green line), and the predicted adult biomass using both NAO and the secular time trend, $log(\widehat{adult\_b_t})$ (orange line). The orange line represents a "counter-

factual" catch trajectory in a world with no NAO variation while holding all other determinants unchanged. The green line examines adult cod dynamics with NAO "turned-on" starting in 1980. The difference between the green and orange lines represent the added contribution of the NAO on adult biomass. To get the percentage contribution in the overall adult biomass decline due to the NAO from period $t = s1$ to $t = s2$, we calculate the following:

$$\text{NAO contribution} = \frac{(\widehat{adult\_b}_{s2} - \widehat{adult\_b}_{s1}) - (\widetilde{adult\_b}_{s2} - \widetilde{adult\_b}_{s1})}{(adult\_b_{s2} - adult\_b_{s1})} \tag{S.4}$$

In practice, due to noisy biomass values, we take the average values over the first 3 and last 3 years of the sample period when applying Eq. S.4. We find that the recent multi-decadal positive phase of the NAO explains 18% of the overall decline in adult biomass in the Gulf of Maine between 1980-2013. For the Georges Bank fishery, that contribution is 9% of the overall decline in adult biomass from 1980-2011.

S.2.5 Modeling NAO effects on cod catch

Cod catch is a function of cod biomass and fishing effort. By examining catch, we can indirectly explore how fishing effort may have historically dampened or enhanced the direct biophysical impact of birth-year NAO on biomass. We model commercial cod catch, $catch_t$ with the following regression model:

$$log(catch_t) = \psi + \sum_{\tau=0}^{L} \delta_\tau NAO_{t-\tau} + \sum_{p=1}^{N} \kappa_p t^p + \mu_t \tag{S.5}$$

where ψ is a constant, δ_τ captures the linear effect of NAO τ periods ago and κ_p captures the effect of a pth-order polynomial time trend. As with Equation S.2, standard errors use the Newey-West adjustment allowing for arbitrary forms of serial correlation and heteroscedasticity in the error terms over an optimally chosen window of time.[7,8] Again, we estimate Equation S.5 separately for the Gulf of Maine and Georges Bank cod fisheries.

Table M shows results for Equation S.5 for the Gulf of Maine fishery in Column (1) and the Georges Bank fishery in Column (2) and corresponds to Panels (A) and (B) of Figure 3 in the main text respectively. The sample period is 1913-2013 for the Gulf of Maine and 1913-2011 for the Georges Bank fishery. Lagged NAO terms up to 20 years prior are included in each model in addition to a 4th-order polynomial time trend. In the Gulf of Maine fishery, we find that a 1-unit increase in NAO is associated with a -3 to -6% change in catch that lasts up to 19 years after the initial event. We find persistent effects of similar magnitude for up to 15 years after a 1-unit increase in NAO for the Georges Bank fishery.

Model selection tests

In Tables N and O we conduct goodness-of-fit and unit-root tests separately for versions of Equation S.5 while varying lag number L (across rows) and the order number of the polynomial time trend N (across columns). For the Gulf of Maine, Table N demonstrates that the AIC statistic generally decreases as the lag number increases up to 20 years prior. The AIC statistic is also minimized for models with longer lag specifications and with higher order polynomial time trends. The Dickey-Fuller Generalized Least Square (DF-GLS) test rejects the presence of a unit root for our benchmark specification with $L = 20$ and $p = 4$. Table O provides diagnostics for the Georges Bank fishery and shows a similar pattern of test results.

S.2.6 Decomposing NAO's effect on cod catch decline since 1980

Panels (C) and (D) of Figure 3 performs a decomposition of NAO's contribution to the overall decline in commercial cod catch since 1980 using a procedure that is identical to that detailed for adult biomass in Section S.2.4. Using Eq. S.4 but for catch, we find that the recent multi-decadal positive phase of the NAO since 1980 explains 32% and 7% of the overall decline in catch in the Gulf of Maine and Georges Bank fisheries respectively.

References

[1] Hurrell, J. W. (1995) Decadal trends in the north atlantic oscillation: Regional temperatures and precipitation. *Science* **269**, 676–679.

[2] Reynolds, R. W, Smith, T. M, Liu, C, Chelton, D. B, Casey, K. S, & Schlax, M. G. (2007) Daily High-Resolution-Blended Analyses for Sea Surface Temperature. *Journal of Climate* **20**, 5473–5496.

[3] NEFSC. (2013) 55th northeast regional stock assessment workshop: Assessment summary report, (U.S. Department of Commerce, NEFSC), Technical Report 41.

[4] NEFSC. (2014) Gulf of maine atlantic cod 2014 assessment update report, (U.S. Department of Commerce, NEFSC), Technical report.

[5] Mayo, R & Col., L. (2006) The 2005 assessment of the gulf of maine atlantic cod stock, (U.S. Department of Commerce, NEFSC), Technical Report 2.

[6] O'Brien, L, Clark, K, Shepherd, N, Traver, M, Tang, J, & Holmes, B. (2006) Assessment of the georges bank atlantic cod stock for 2005., (U.S. Department of Commerce, NEFSC), Technical Report 10.

[7] Newey, W. K & West, K. D. (1987) A simple, positive semi-definite, heteroskedasticity and autocorrelation consistent covariance matrix. *Econometrica* **55**, 703–708.

[8] Newey, W. K & West, K. D. (1994) Automatic lag selection in covariance matrix estimation. *The Review of Economic Studies* **61**, pp. 631–653.

[9] Akaike, H. (1974) A new look at the statistical model identification. *Automatic Control, IEEE Transactions on* **19**, 716–723.

[10] Elliott, G, Rothenberg, T. J, & Stock, J. H. (1996) Efficient tests for an autoregressive unit root. *Econometrica* **64**, 813–836.

[11] Granger, C & Newbold, P. (1974) Spurious regressions in econometrics. *Journal of Econometrics* **2**, 111 – 120.

[12] Fan, J & Gijbels, I. (1996) *Local Polynomial Modelling and Its Applications.* (Chapman & Hall, London).

[13] Stige, L. C, Otterson, G, Brander, K, Chan, K.-S, & Stenseth, N. C. (2006) Cod and climate: effect of the north atlantic oscillation on recruitment in the north atlantic. *Marine Ecological Progress Series* **325**, 227–241.

[14] Angrist, J. D & Pischke, J.-S. (2008) *Mostly Harmless Econometrics: An Empiricist's Companion.* (Princeton University Press).

[15] Hsiang, S. M & Jina, A. S. (2014) The causal effect of environmental catastrophe on long-run economic growth: Evidence from 6,700 cyclones, (National Bureau of Economic Research), Working Paper 20352.

S.3 FIGURES

Figure A: Birth-year NAO effect using SLP and PC-based winter NAO indices

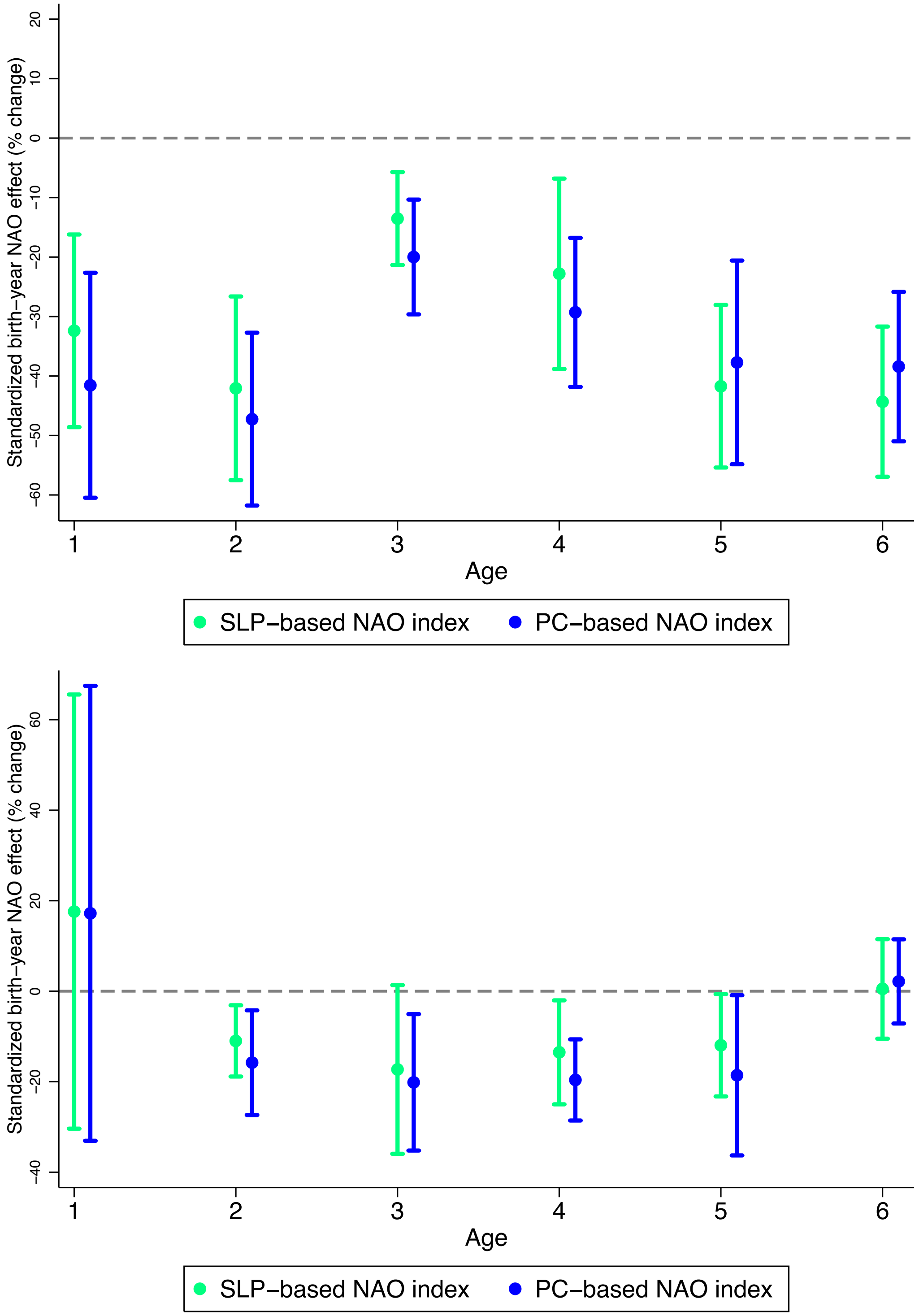

Notes: Top and bottom panels show regression coefficients representing the effect of a 1-unit increase in birth-year NAO on a cod cohort as it matures from age 1 to 6 for the Gulf of Maine and Georges Bank stocks, respectively. Each coefficient comes from a separate multiple regression model (see Eq. S.2). Green shows effects using the station NAO index based on sea-level pressure (SLP) differences. Blue shows effects using a principal-component based NAO index. Both NAO indices standardized to zero mean and unit variance. 90% confidence interval shown.

Figure B: Testing for nonlinearities in birth-year NAO effect

A) Gulf of Maine

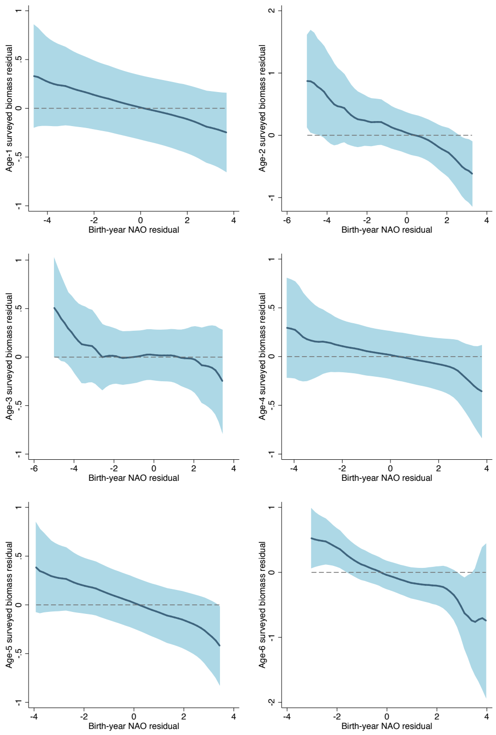

B) Georges Bank

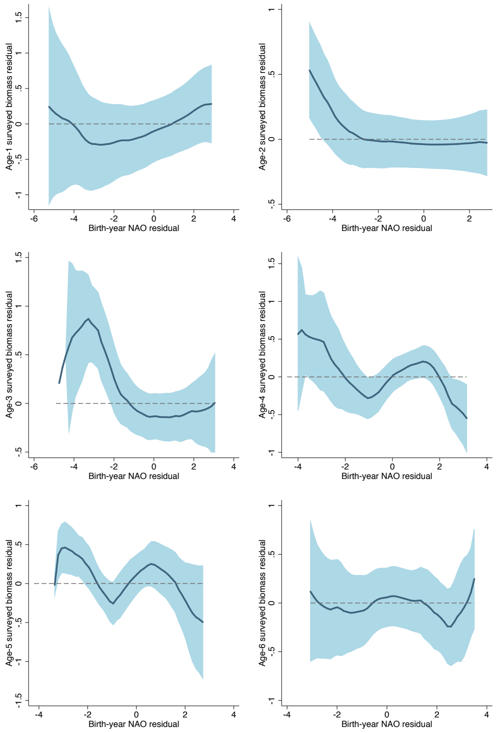

Notes: Plots test the linearity of birth-year NAO effect on age-specific surveys biomass for ages 1-6 Gulf of Maine (Panel A) and Georges Bank (Panel B). Both variables are first regressed on post-birth-year NAO, birth-year spawning stock in levels and logs, and a 3rd-order polynomial time trend (see Eq. S.2). Residuals are then fitted using a local polynomial regression with an Epanechnikov kernel and "rule-of-thumb" bandwidth.[12] 90% confidence intervals shown.

Figure C: Testing for time-invariant NAO-recruitment effect

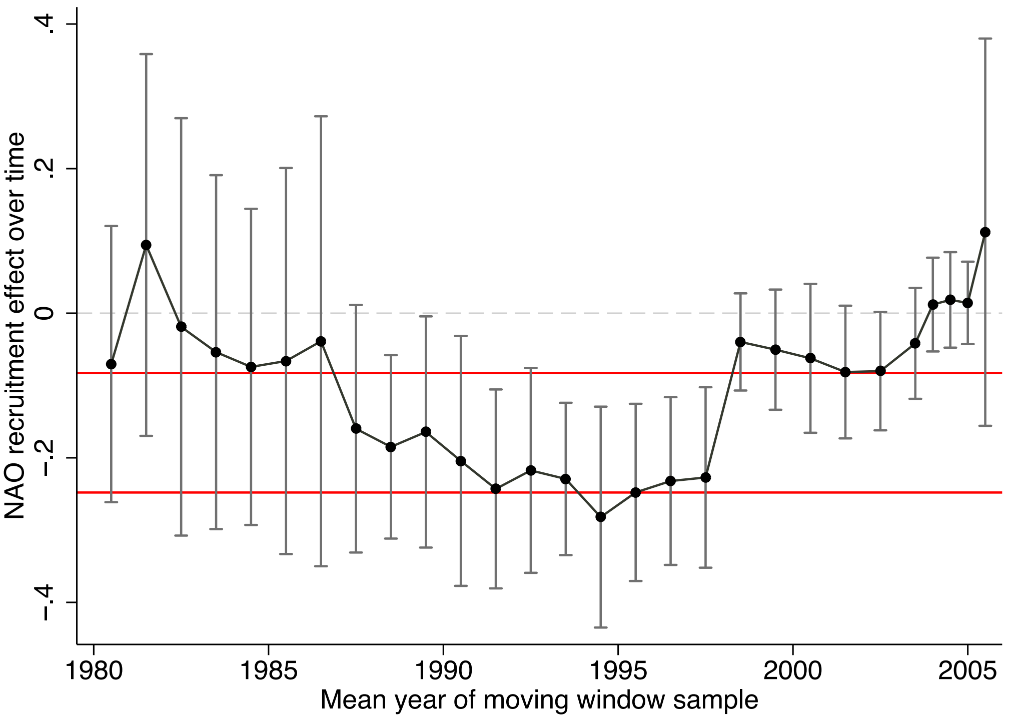

Notes: Plots birth-year NAO recruitment (i.e. age-1 cod) effect for Gulf of Maine using a 20-year moving window. Mean year of each estimation window shown on x-axis. Model specification from Eq. 2 of main text with 3rd-order polynomial time trend. Serial correlation and heteroscedasticity robust Newey-West standard errors with optimal bandwidth. 90% confidence intervals shown. Horizontal red lines show 90% confidence interval for the full sample effect.

S.4 TABLES

Table A: Trend selection for winter NAO effects on sea surface temperatures in Gulf of Maine

	Dep. var. is average winter SST (DJFM)			
	(1)	(2)	(3)	(4)
NAO_t	0.0206	0.0442**	0.0453***	0.0458**
	[0.0325]	[0.0188]	[0.0154]	[0.0200]
Observations	32	32	32	32
Sample period	1982-2013	1982-2013	1982-2013	1982-2013
Number of trends	1	2	3	4
AIC	51.85	36.98	36.91	36.99
Newey-West bandwidth	16	16	16	16

Notes: Each column shows the coefficient from a time-series regression of winter SST (DJFM), in degrees Celsius, averaged over grid-cells in the Gulf of Maine fishery on NAO. Order of polynomial time trend varies across columns. Serial correlation and heteroscedasticity robust Newey-West standard errors with optimal bandwidth. *** p<0.01, ** p<0.05, * p<0.1

Table B: Trend selection for winter NAO effects on sea surface temperatures in Georges Bank

	Dep. var. is average winter SST (DJFM)			
	(1)	(2)	(3)	(4)
NAO_t	0.0597	0.0849***	0.0808***	0.0813**
	[0.0460]	[0.0320]	[0.0308]	[0.0362]
Observations	32	32	32	32
Sample period	1982-2013	1982-2013	1982-2013	1982-2013
Number of time trends	1	2	3	4
AIC	70.68	63.13	62.66	62.59
Newey-West bandwidth	16	16	16	16

Notes: Each column shows the coefficient from a time-series regression of winter SST (DJFM), in degrees Celsius, averaged over grid-cells in the George's Bank fishery on NAO. Order of polynomial time trend varies across columns. Serial correlation and heteroscedasticity robust Newey-West standard errors with optimal bandwidth. *** p<0.01, ** p<0.05, * p<0.1

Table C: Effects of NAO on age-specific biomass in Gulf of Maine

	Dep. var. is log cohort-specific spring cod biomass											
	(1)	(2)	(3)	(4)	(5)	(6)	(7)	(8)	(9)	(10)	(11)	(12)
	age=1	age=1	age=2	age=2	age=3	age=3	age=4	age=4	age=5	age=5	age=6	age=6
mean biomass (in kg)	0.0200	0.0200	0.200	0.200	0.730	0.730	1.320	1.320	1.230	1.230	0.880	0.880
NAO_t	-0.0258	-0.0234	0.115*	0.118*	0.0821	0.0980*	-0.00496	-0.00873	0.00913	-0.0185	-0.0744	-0.100
	[0.0517]	[0.0519]	[0.0673]	[0.0684]	[0.0662]	[0.0564]	[0.110]	[0.119]	[0.0610]	[0.101]	[0.0768]	[0.0854]
NAO_{t-1}	**-0.165*** **	-0.164***	0.0527	0.0526	0.0784***	0.0857***	0.0499	0.0500	-0.0974	-0.0971	-0.107*	-0.118**
	[0.0503]	[0.0394]	[0.0871]	[0.0851]	[0.0219]	[0.0198]	[0.0621]	[0.0729]	[0.0971]	[0.0630]	[0.0558]	[0.0526]
NAO_{t-2}			**-0.215*** **	-0.204**	0.0299	0.0658	0.0176	0.00711	0.110***	0.0760	-0.158*	-0.198**
			[0.0488]	[0.0697]	[0.0781]	[0.122]	[0.0456]	[0.113]	[0.0411]	[0.0902]	[0.0903]	[0.100]
NAO_{t-3}					**-0.0690*** **	-0.0282	0.0317	0.0200	-0.0270	-0.0681	0.0236	-0.0180
					[0.0243]	[0.0929]	[0.0392]	[0.126]	[0.0359]	[0.0822]	[0.0416]	[0.0547]
NAO_{t-4}							**-0.116*** **	-0.117***	0.0325	0.0245	-0.0293	-0.0377
							[0.0496]	[0.0448]	[0.0875]	[0.111]	[0.0609]	[0.0643]
NAO_{t-5}									**-0.213*** **	-0.179*	-0.0298	-0.00594
									[0.0424]	[0.106]	[0.0784]	[0.0806]
NAO_{t-6}											**-0.226*** **	-0.231***
											[0.0391]	[0.0562]
$catch_{t-1}$		-0.0634		-0.0244		-0.0879		0.0265		0.101		0.0906*
		[0.0883]		[0.0564]		[0.154]		[0.195]		[0.151]		[0.0483]
adult $biomass_{t-age}$	0.170	0.171	0.212	0.212	0.164***	0.165**	-0.000259	-0.00216	0.0770	0.0825	0.191	0.242
	[0.191]	[0.179]	[0.175]	[0.180]	[0.0566]	[0.0770]	[0.149]	[0.174]	[0.167]	[0.182]	[0.178]	[0.160]
ln[adult $biomass_{t-age}$]	-0.618	-0.592	-0.759	-0.751	-0.943***	-0.977**	-0.0211	0.0204	-0.200	-0.131	-0.636	-0.864
	[0.689]	[0.669]	[0.879]	[0.898]	[0.358]	[0.390]	[0.539]	[0.403]	[0.889]	[0.808]	[0.910]	[0.858]
Observations	43	43	42	42	41	41	40	40	39	39	38	38
Sample period	1971-2013	1971-2013	1972-2013	1972-2013	1973-2013	1973-2013	1974-2013	1974-2013	1975-2013	1975-2013	1976-2013	1976-2013
Number of trends	3	3	3	3	3	3	3	3	3	3	3	3
Newey-West bandwidth	17	17	17	17	2	11	17	17	17	17	17	9

Notes: Each column shows coefficients from a time-series regression model of log cohort-specific spring cod biomass on current and past NAO, adult biomass (ages 2 and up) from the spawning year of that cohort, and trend terms. Some models additionally include control for previous year catch. Coefficients shaded in gray capture birth-year NAO effects. Coefficients in **bold** correspond to coefficients shown in Figure 2 of the main text. Serial correlation and heteroscedasticity robust Newey-West standard errors with optimal bandwidth. *** p<0.01, ** p<0.05, * p<0.1

Table D: Trend selection tests for age-specific biomass models in Gulf of Maine

	Number of trend terms				
	(1)	(2)	(3)	(4)	(5)
		Panel (1) Dep. var. is age 1 cohort biomass			
NAO_{t-1}	-0.147**	-0.162***	**-0.165*** **	-0.165***	-0.150****
	[0.0574]	[0.0527]	[0.0502]	[0.0502]	[0.0522]
Dickey-Fuller p-value	***	***	***	***	–
AIC	150.9	152.8	152.5	152.5	151.8
		Panel (2) Dep. var. is age 2 cohort biomass			
NAO_{t-2}	-0.210***	-0.227***	**-0.215*** **	-0.216***	-0.203***
	[0.0392]	[0.0620]	[0.0488]	[0.0490]	[0.0563]
Dickey-Fuller p-value	–	–	–	–	***
AIC	139.5	141.4	142.0	140.3	136.7
		Panel (3) Dep. var. is age 3 cohort biomass			
NAO_{t-3}	-0.0669**	-0.0759***	**-0.0690*** **	-0.0696***	-0.0633**
	[0.0330]	[0.0245]	[0.0242]	[0.0252]	[0.0323]
Dickey-Fuller p-value	***	***	***	***	***
AIC	117.4	119.4	118.7	118.8	117.2
		Panel (4) Dep. var. is age 4 cohort biomass			
NAO_{t-4}	-0.0969***	-0.116**	**-0.116** **	-0.116**	-0.0761*
	[0.0336]	[0.0495]	[0.0497]	[0.0501]	[0.0452]
Dickey-Fuller p-value	–	–	–	–	***
AIC	115.7	117.4	117.4	117.3	116.0
		Panel (5) Dep. var. is age 5 cohort biomass			
NAO_{t-5}	-0.217***	-0.213***	**-0.213*** **	-0.212***	-0.211***
	[0.0370]	[0.0424]	[0.0424]	[0.0425]	[0.0437]
Dickey-Fuller p-value	**	*	**	–	–
AIC	123.3	125.3	125.0	125.0	124.9
		Panel (6) Dep. var. is age 6 cohort biomass			
NAO_{t-6}	-0.156***	-0.221***	**-0.226*** **	-0.223***	-0.220***
	[0.0435]	[0.0545]	[0.0389]	[0.0394]	[0.0399]
Dickey-Fuller p-value	–	–	–	–	–
AIC	113.6	113.7	102.3	102.0	101.7

Notes: Each column and row shows statistics from separate time-series regressions. Each panel uses a different age cohort biomass as the dependent variable. Order of polynomial time trend varies across columns. Table shows birth-year NAO effects with coefficients in **bold** corresponding to that shown in Figure 2 of the main text. Model AIC statistic and p-value from Dickey-Fuller tests model residual against the presence of a unit root also shown. *** p<0.01, ** p<0.05, * p<0.1, – p>0.1

Table E: Lag selection tests for age-specific biomass models in Gulf of Maine

	birth-year NAO only	Additional pre-birth-year NAO terms (1)	(2)	(3)	(4)	(5)	(6)
				Panel (1) Dep. var. is age 1 cohort biomass			
NAO_{t-1}	-0.166***	**-0.165*****	-0.147**	-0.163**	-0.190***	-0.191***	-0.155**
	[0.0523]	[0.0502]	[0.0623]	[0.0721]	[0.0676]	[0.0626]	[0.0638]
Dickey-Fuller p-value	**	***	***	***	*	*	***
AIC	152.6	152.5	155.6	156.9	155.9	157.9	158.3
				Panel (2) Dep. var. is age 2 cohort biomass			
NAO_{t-2}	-0.235***		**-0.215*****	-0.209***	-0.242***	-0.243***	-0.284***
	[0.0321]		[0.0488]	[0.0486]	[0.0411]	[0.0556]	[0.0734]
Dickey-Fuller p-value	–		–	–	***	***	***
AIC	140.3		142.0	143.9	139.7	141.7	141.1
				Panel (3) Dep. var. is age 3 cohort biomass			
NAO_{t-3}	-0.0905***			**-0.0690*****	-0.0657**	-0.0740**	-0.0663**
	[0.0276]			[0.0242]	[0.0284]	[0.0340]	[0.0312]
Dickey-Fuller p-value	***			***	***	***	***
AIC	117.5			118.7	120.6	124.1	125.6
				Panel (4) Dep. var. is age 4 cohort biomass			
NAO_{t-4}	-0.123***				**-0.116****	-0.103**	-0.155***
	[0.0275]				[0.0497]	[0.0482]	[0.0357]
Dickey-Fuller p-value	–				–	–	–
AIC	112.1				117.4	118.7	114.0
				Panel (5) Dep. var. is age 5 cohort biomass			
NAO_{t-5}	-0.199***					**-0.213*****	-0.204***
	[0.0706]					[0.0424]	[0.0463]
Dickey-Fuller p-value	*					**	–
AIC	120.0					125.0	126.9
				Panel (6) Dep. var. is age 6 cohort biomass			
NAO_{t-6}	-0.170**						**-0.226*****
	[0.0667]						[0.0389]
Dickey-Fuller p-value	–						–
AIC	102.1						102.3

Notes: Each column and row shows statistics from separate time-series regressions. Each panel uses a different age cohort biomass as dependent variable. First column includes only birth-year NAO term. Each subsequent column further includes an additional lagged NAO term. All models include a 3rd-order polynomial time trend. Table shows birth-year NAO effects with coefficients in **bold** corresponding to that shown in Figure 2 of the main text. Model AIC statistic and p-value from Dickey-Fuller tests model residual against the presence of a unit root also shown. *** p<0.01, ** p<0.05, * p<0.1, – p>0.1

Table F: Intergenerational effects of NAO on age-specific biomass in Gulf of Maine

	Dep. var. is log age-specific spring cod biomass			
	(1)	(2)	(3)	(4)
	Age 1	Age 2	Age 3	Age 4
mean biomass (in kg)	0.0200	0.200	0.730	1.320
NAO_t	-0.165**	0.0166	0.108	-0.0932
	[0.0788]	[0.103]	[0.0724]	[0.0757]
NAO_{t-1}	-0.155**	0.0364	0.0444	0.110***
	[0.0641]	[0.0860]	[0.0336]	[0.0268]
NAO_{t-2}	-0.187	-0.284***	0.0534	-0.101**
	[0.135]	[0.0735]	[0.0794]	[0.0433]
NAO_{t-3}	-0.0721	-0.0139	-0.0663**	0.00389
	[0.108]	[0.0799]	[0.0311]	[0.0170]
NAO_{t-4}	-0.220**	-0.248***	0.00370	-0.155***
	[0.0977]	[0.0886]	[0.0560]	[0.0357]
NAO_{t-5}	0.0225	0.00923	-0.0712	0.00291
	[0.0724]	[0.0864]	[0.0545]	[0.0370]
NAO_{t-6}	-0.154***	-0.0795	0.0558	-0.249***
	[0.0554]	[0.113]	[0.0829]	[0.0580]
Observations	43	42	41	40
Sample period	1971-2013	1972-2013	1973-2013	1974-2013
Number of trends	3	3	3	3
Newey-West bandwidth	17	6	13	17

Notes: Each column shows coefficients from a time-series regression of log age-specific spring cod biomass on current and past NAO. Coefficients shaded in gray capture birth-year NAO effects. All models include a 3rd-order polynomial time trend. Serial correlation and heteroscedasticity robust Newey-West standard errors with optimal bandwidth. *** p<0.01, ** p<0.05, * p<0.1

Table G: Effects of NAO on age-specific biomass in Georges Bank

	Dep. var. is log cohort-specific spring cod biomass											
	(1)	(2)	(3)	(4)	(5)	(6)	(7)	(8)	(9)	(10)	(11)	(12)
	age=1	age=1	age=2	age=2	age=3	age=3	age=4	age=4	age=5	age=5	age=6	age=6
mean biomass (in kg)	0.120	0.120	0.790	0.790	2.410	2.410	2.760	2.760	2.680	2.680	1.290	1.290
NAO_t	0.0649	0.00288	0.0348	0.0702*	-0.0430***	-0.0427**	-0.0442	-0.00377	-0.0882*	-0.0510	-0.0477	-0.0904
	[0.0408]	[0.0788]	[0.0351]	[0.0396]	[0.0158]	[0.0196]	[0.0593]	[0.0515]	[0.0456]	[0.0462]	[0.0551]	[0.0789]
NAO_{t-1}	**0.0898**	0.0643	0.0601	0.0721	0.0964**	0.0964**	-0.0423	-0.0330	-0.0387	-0.0373	-0.0755	-0.0911*
	[0.148]	[0.159]	[0.0491]	[0.0492]	[0.0485]	[0.0480]	[0.0589]	[0.0623]	[0.0576]	[0.0565]	[0.0538]	[0.0490]
NAO_{t-2}			**-0.0560****	-0.0332	-0.0605	-0.0602	-0.0657**	-0.0413*	-0.149***	-0.132*	-0.118	-0.110
			[0.0230]	[0.0300]	[0.0632]	[0.0705]	[0.0315]	[0.0234]	[0.0503]	[0.0707]	[0.102]	[0.0990]
NAO_{t-3}					**-0.0883**	-0.0882	-0.0816**	-0.0637*	-0.0947**	-0.0887*	-0.0910	-0.0953
					[0.0586]	[0.0559]	[0.0377]	[0.0326]	[0.0478]	[0.0476]	[0.0997]	[0.0930]
NAO_{t-4}							**-0.0690***	-0.0860**	-0.0901	-0.103	-0.104	-0.0593
							[0.0357]	[0.0374]	[0.0713]	[0.0787]	[0.0813]	[0.0651]
NAO_{t-5}									**-0.0609***	-0.0918	-0.112**	-0.0711
									[0.0354]	[0.0588]	[0.0448]	[0.0471]
NAO_{t-6}											**0.00256**	0.0654***
											[0.0333]	[0.0253]
$catch_{t-1}$		0.0.0518*		-0.0259**		-0.000191		-0.0276**		-0.0266		0.0416**
		[0.0287]		[0.0116]		[0.00832]		[0.0133]		[0.0250]		[0.0166]
adult $biomass_{t-age}$	-0.0305***	-0.0365***	-0.0441***	-0.0443***	-0.0214***	-0.0214***	-0.0161***	-0.0171***	-0.0499***	-0.0533***	-0.0398***	-0.0358***
	[0.0118]	[0.0116]	[0.00604]	[0.00538]	[0.00629]	[0.00765]	[0.00421]	[0.00564]	[0.00817]	[0.00770]	[0.00993]	[0.00906]
ln[adult $biomass_{t-age}$]	1.069*	0.921**	0.725***	0.801***	0.201	0.202	0.0683	0.0534	0.479**	0.477**	0.597**	0.479*
	[0.562]	[0.454]	[0.202]	[0.181]	[0.158]	[0.180]	[0.146]	[0.153]	[0.233]	[0.216]	[0.292]	[0.261]
Observations	33	33	32	32	31	31	30	30	29	29	28	28
Sample period	1979-2011	1979-2011	1980-2011	1980-2011	1981-2011	1981-2011	1982-2011	1982-2011	1983-2011	1983-2011	1984-2011	1984-2011
Number of trends	3	3	3	3	3	3	3	3	3	3	3	3
Newey-West bandwidth	16	16	16	16	16	16	16	16	16	16	16	16

Notes: Each column shows coefficients from a time-series regression model of log cohort-specific spring cod biomass on current and past NAO, adult biomass (ages 2 and up) from the spawning year of that cohort, and trend terms. Some models additionally include control for previous year catch. Coefficients shaded in gray capture birth-year NAO effects. Coefficients in **bold** correspond to coefficients shown in Figure 2 of the main text. Serial correlation and heteroscedasticity robust Newey-West standard errors with optimal bandwidth. *** p<0.01, ** p<0.05, * p<0.1

Table H: Trend selection tests for age-specific biomass models in Georges Bank

	Number of trend terms				
	(1)	(2)	(3)	(4)	(5)
	Panel (1) Dep. var. is age 1 cohort biomass				
NAO_{t-1}	0.0606	0.0992	**0.0898**	0.0901	0.0906
	[0.109]	[0.147]	[0.149]	[0.149]	[0.149]
Dickey-Fuller p-value	***	***	***	***	***
AIC	126.1	127.1	126.2	126.2	126.2
	Panel (2) Dep. var. is age 2 cohort biomass				
NAO_{t-2}	-0.0721***	-0.0322	**-0.0560****	-0.0570**	-0.0578**
	[0.0152]	[0.0271]	[0.0239]	[0.0256]	[0.0282]
Dickey-Fuller p-value	*	**	**	**	**
AIC	77.03	72.47	70.02	69.94	69.87
	Panel (3) Dep. var. is age 3 cohort biomass				
NAO_{t-3}	-0.123*	-0.101*	**-0.0883**	-0.0881	-0.0879
	[0.0682]	[0.0594]	[0.0584]	[0.0581]	[0.0575]
Dickey-Fuller p-value	**	–	–	–	–
AIC	74.37	74.79	76.30	74.31	74.34
	Panel (4) Dep. var. is age 4 cohort biomass				
NAO_{t-4}	-0.114***	-0.102***	**0.0811**	-0.0681	-0.0674
	[0.0284]	[0.0271]	[0.0649]	[0.0451]	[0.0413]
Dickey-Fuller p-value	–	–	–	–	–
AIC	75.70	77.55	77.22	74.67	74.77
	Panel (5) Dep. var. is age 5 cohort biomass				
NAO_{t-5}	-0.0400	-0.0722***	**-0.0609***	-0.0596*	-0.0583
	[0.0274]	[0.0271]	[0.0358]	[0.0344]	[0.0431]
Dickey-Fuller p-value	***	***	***	***	***
AIC	82.58	83.87	84.48	80.36	82.24
	Panel (6) Dep. var. is age 6 cohort biomass				
NAO_{t-6}	0.0130	-0.0444*	**-0.112*****	0.00583	0.00912
	[0.0253]	[0.0260]	[0.0431]	[0.0362]	[0.700]
Dickey-Fuller p-value	–	**	–	–	–
AIC	87.33	87.39	85.62	85.52	83.43

Notes: Each column and row shows statistics from separate time-series regressions. Each panel uses a different age cohort biomass as the dependent variable. Order of polynomial time trend varies across columns. Table shows birth-year NAO effects with coefficients in **bold** corresponding to that shown in Figure 2 of the main text. Model AIC statistic and p-value from Dickey-Fuller tests model residual against the presence of a unit root also shown. *** $p<0.01$, ** $p<0.05$, * $p<0.1$, – $p>0.1$

Table I: Lag selection tests for age-specific biomass models in Georges Bank

		Additional pre-birth-year NAO terms					
	birth-year NAO only	(1)	(2)	(3)	(4)	(5)	(6)
				Panel (1) Dep. var. is age 1 cohort biomass			
NAO_{t-1}	0.0953	**0.0898**	0.0787	0.0647	0.0262	0.0442	0.0381
	[0.152]	[0.149]	[0.148]	[0.147]	[0.158]	[0.148]	[0.151]
Dickey-Fuller p-value	***	***	***	***	–	–	–
AIC	126.5	126.2	127.4	126.6	127.6	129.4	133.4
				Panel (2) Dep. var. is age 2 cohort biomass			
NAO_{t-2}	-0.0590**		**-0.0560****	-0.0559*	-0.0705**	-0.0708**	-0.0527
	[0.0294]		[0.0239]	[0.0299]	[0.0318]	[0.0303]	[0.0361]
Dickey-Fuller p-value	**		**	**	**	**	**
AIC	69.91		70.02	69.64	71.94	73.86	77.51
				Panel (3) Dep. var. is age 3 cohort biomass			
NAO_{t-3}	-0.102*			**-0.0883**	-0.0907	-0.0989*	-0.0973*
	[0.0569]			[0.0584]	[0.0593]	[0.0562]	[0.0568]
Dickey-Fuller p-value	–			–	–	–	–
AIC	71.45			76.30	77.99	79.08	79.03
				Panel (4) Dep. var. is age 4 cohort biomass			
NAO_{t-4}	-0.0357				**-0.0690***	-0.0710	-0.0767*
	[0.0400]				[0.0357]	[0.0437]	[0.0405]
Dickey-Fuller p-value	***				***	***	***
AIC	69.42				74.57	77.22	79.10
				Panel (5) Dep. var. is age 5 cohort biomass			
NAO_{t-5}	-0.0404					**-0.0609***	-0.0651
	[[0.0362]					[0.0358]	[0.0434]
Dickey-Fuller p-value	***					***	***
AIC	77.59					84.48	86.37
				Panel (6) Dep. var. is age 6 cohort biomass			
NAO_{t-6}	0.0574*						**0.00256**
	[0.0315]						[0.0343]
Dickey-Fuller p-value	**						–
AIC	77.90						87.62

Notes: Each column and row shows statistics from separate time-series regressions. Each panel uses a different age cohort biomass as dependent variable. First column includes only birth-year NAO term. Each subsequent column further includes an additional lagged NAO term. All models include a 3rd-order polynomial time trend. Table shows birth-year NAO effects with coefficients in **bold** corresponding to that shown in Figure 2 of the main text. Model AIC statistic and p-value from Dickey-Fuller tests model residual against the presence of a unit root also shown. *** p<0.01, ** p<0.05, * p<0.1, – p>0.1

Table J: Intergenerational effects of NAO on age-specific biomass in Georges Bank

	Dep. var. is log age-specific spring cod biomass			
	(1)	(2)	(3)	(4)
	Age 1	Age 2	Age 3	Age 4
mean biomass (in kg)	0.120	0.790	2.410	2.760
NAO_t	-0.0169	0.0337	-0.0345	-0.0707**
	[0.0914]	[0.0240]	[0.0293]	[0.0345]
NAO_{t-1}	0.0381	0.0370	0.0688	-0.0116
	[0.151]	[0.0591]	[0.0419]	[0.0305]
NAO_{t-2}	0.0800	-0.0527	-0.0694	-0.0814***
	[0.0745]	[0.0396]	[0.0624]	[0.0161]
NAO_{t-3}	-0.133	0.0298	-0.0973*	-0.0661*
	[0.150]	[0.0422]	[0.0568]	[0.0399]
NAO_{t-4}	-0.232	-0.0693	-0.0352	-0.0767*
	[0.179]	[0.0628]	[0.0360]	[0.0394]
NAO_{t-5}	0.0519	-0.0212	-0.0658	0.0831
	[0.121]	[0.0688]	[0.0492]	[0.0768]
NAO_{t-6}	0.0285	0.0387	0.0174	-0.0244
	[0.0358]	[0.0513]	[0.0326]	[0.0917]
Observations	33	32	31	30
Sample period	1979-2011	1980-2011	1981-2011	1982-2011
Number of trends	3	3	3	3
Newey-West bandwidth	16	16	16	16

Notes: Each column shows coefficients from a time-series regression of log age-specific spring cod biomass on current and past NAO. Coefficients shaded in gray capture birth-year NAO effects. All models include a 3rd-order polynomial time trend. Serial correlation and heteroscedasticity robust Newey-West standard errors with optimal bandwidth. *** p<0.01, ** p<0.05, * p<0.1

Table K: Effects of NAO on adult cod biomass in Gulf of Maine

	Dep. var. is log adult (ages 2-6) surveyed biomass							
	(1)	(2)	(3)	(4)	(5)	(6)	(7)	(8)
mean biomass (kg)	4.350	4.350	4.350	4.350	4.350	4.350	4.350	4.350
NAO_t	0.0256	0.0255	-0.0181	-0.0179	-0.0181	-0.0160	-0.0178	-0.0167
	[0.0431]	[0.0497]	[0.0390]	[0.0421]	[0.0380]	[0.0383]	[0.0389]	[0.0393]
NAO_{t-1}	0.0338	0.0338	0.0190	0.0191	0.0168	0.0158	0.0181	0.0176
	[0.0349]	[0.0358]	[0.0361]	[0.0364]	[0.0397]	[0.0395]	[0.0397]	[0.0403]
NAO_{t-2}	0.0362	0.0360	-0.0146	-0.0142	-0.0147	-0.0112	-0.0142	-0.0123
	[0.0272]	[0.0321]	[0.0255]	[0.0255]	[0.0247]	[0.0235]	[0.0253]	[0.0245]
NAO_{t-3}	0.0483***	0.0482**	0.0199	0.0203	0.0202	0.0243	0.0202	0.0225
	[0.0131]	[0.0201]	[0.0184]	[0.0172]	[0.0198]	[0.0211]	[0.0192]	[0.0215]
NAO_{t-4}	-0.0365	-0.0367	-0.0765***	-0.0759**	-0.0772***	-0.0726**	-0.0766***	-0.0741**
	[0.0249]	[0.0447]	[0.0274]	[0.0384]	[0.0289]	[0.0360]	[0.0293]	[0.0354]
NAO_{t-5}	-0.0452	-0.0452	-0.0601*	-0.0602	-0.0616*	-0.0641*	-0.0606*	-0.0621*
	[0.0387]	[0.0416]	[0.0364]	[0.0388]	[0.0327]	[0.0367]	[0.0335]	[0.0370]
NAO_{t-6}	-0.0575	-0.0576	-0.108**	-0.108**	-0.108*	-0.107*	-0.108*	-0.107**
	[0.0534]	[0.0515]	[0.0541]	[0.0535]	[0.0555]	[0.0552]	[0.0551]	[0.0548]
$catch_{t-1}$		0.00334		-0.00837		-0.0804		-0.0445
		[0.342]		[0.214]		[0.147]		[0.152]
Observations	44	44	44	44	44	44	44	44
Sample period	1970-2013	1970-2013	1970-2013	1970-2013	1970-2013	1970-2013	1970-2013	1970-2013
Number of trends	1	1	2	2	3	3	4	4
Newey-West bandwidth	17	17	17	17	17	17	17	17

Notes: Each column shows coefficients from a time-series regression model of log adult (ages 2-6) surveyed biomass on current and past NAO and time-trend terms. Models in even numbered columns additionally include control for previous year catch. Serial correlation and heteroscedasticity robust Newey-West standard errors with optimal bandwidth. *** p<0.01, ** p<0.05, * p<0.1

Table L: Effects of NAO on adult cod biomass in Georges Bank

	Dep. var. is log adult (ages 2-6) surveyed biomass							
	(1)	(2)	(3)	(4)	(5)	(6)	(7)	(8)
mean biomass (kg)	9.930	9.930	9.930	9.930	9.930	9.930	9.930	9.930
NAO_t	-0.0181	0.00103	-0.0366*	-0.0131	-0.0311	-0.0133	-0.0303	-0.0133
	[0.0223]	[0.0186]	[0.0205]	[0.0180]	[0.0208]	[0.0165]	[0.0210]	[0.0159]
NAO_{t-1}	0.00294	0.0161	-0.0110	0.00417	-0.00822	0.00390	-0.00803	0.00382
	[0.0261]	[0.0255]	[0.0261]	[0.0243]	[0.0269]	[0.0247]	[0.0283]	[0.0257]
NAO_{t-2}	-0.0730	-0.0682	-0.0886*	-0.0909**	-0.0834**	-0.0896**	-0.0823**	-0.0890**
	[0.0473]	[0.0474]	[0.0461]	[0.0425]	[0.0417]	[0.0411]	[0.0406]	[0.0394]
NAO_{t-3}	-0.0975*	-0.0888	-0.107**	-0.0976	-0.103*	-0.0971*	-0.102*	-0.0968*
	[0.0531]	[0.0544]	[0.0544]	[0.0604]	[0.0547]	[0.0589]	[0.0544]	[0.0550]
NAO_{t-4}	-0.0767***	-0.0757***	-0.0920***	-0.102**	-0.0891***	-0.100**	-0.0882***	-0.0998***
	[0.0269]	[0.0285]	[0.0276]	[0.0415]	[0.0290]	[0.0420]	[0.0296]	[0.0377]
NAO_{t-5}	-0.0593	-0.0648	-0.0671	-0.0837*	-0.0738*	-0.0843*	-0.0739*	-0.0843*
	[0.0413]	[0.0420]	[0.0409]	[0.0456]	[0.0442]	[0.0453]	[0.0432]	[0.0466]
NAO_{t-6}	-0.0165	-0.0317	-0.0299	-0.0697***	-0.0330	-0.0679***	-0.0324	-0.0672***
	[0.0323]	[0.0287]	[0.0346]	[0.0224]	[0.0311]	[0.0212]	[0.0311]	[0.0219]
$catch_{t-1}$		-0.243**		-0.476**		-0.446		-0.436
		[0.113]		[0.242]		[0.300]		[0.276]
Observations	34	34	34	34	34	34	34	34
Sample period	1978-2011	1978-2011	1978-2011	1978-2011	1978-2011	1978-2011	1978-2011	1978-2011
Number of trends	1	1	2	2	3	3	4	4
Newey-West bandwidth	16	16	16	12	16	13	16	16

Notes: Each column shows coefficients from a time-series regression model of log adult (ages 2-6) surveyed biomass on current and past NAO and time-trend terms. Models in even numbered columns additionally include control for previous year catch. Serial correlation and heteroscedasticity robust Newey-West standard errors with optimal bandwidth. *** p<0.01, ** p<0.05, * p<0.1

Table M: Effects of NAO on commercial cod catch for both fisheries

	Dep. var. is log commercial catch	
	(1)	(2)
	GOM	GB
NAO_t	0.0318*	-0.0245
	[0.0180]	[0.0226]
NAO_{t-1}	0.000101	-0.0387***
	[0.0178]	[0.00970]
NAO_{t-2}	0.00948	-0.0376***
	[0.0179]	[0.0104]
NAO_{t-3}	0.00426	-0.0560***
	[0.0244]	[0.0151]
NAO_{t-4}	-0.0282**	-0.0296**
	[0.0120]	[0.0123]
NAO_{t-5}	-0.0546***	-0.0488**
	[0.0161]	[0.0211]
NAO_{t-6}	-0.0439***	-0.0387**
	[0.0122]	[0.0176]
NAO_{t-7}	-0.0407***	-0.0130
	[0.0156]	[0.0189]
NAO_{t-8}	-0.0268**	-0.00679
	[0.0123]	[0.0187]
NAO_{t-9}	-0.0519***	-0.0228
	[0.0162]	[0.0145]
NAO_{t-10}	-0.0377*	-0.0261*
	[0.0204]	[0.0135]
NAO_{t-11}	-0.0385***	-0.0343***
	[0.0127]	[0.0128]
NAO_{t-12}	-0.0458***	-0.0374***
	[0.00779]	[0.0128]
NAO_{t-13}	-0.0647***	-0.0377***
	[0.00854]	[0.0110]
NAO_{t-14}	-0.0461***	-0.0375***
	[0.00900]	[0.0136]
NAO_{t-15}	-0.0292***	-0.0443***
	[0.00896]	[0.0140]
NAO_{t-16}	-0.0226**	-0.00173
	[0.0115]	[0.0131]
NAO_{t-17}	-0.0204*	0.0119
	[0.0121]	[0.0188]
NAO_{t-18}	-0.0362***	0.00593
	[0.0124]	[0.0152]
NAO_{t-19}	-0.0362***	0.00219
	[0.0140]	[0.0190]
NAO_{t-20}	0.0167	0.0100
	[0.0133]	[0.0237]
Observations	101	99
Sample period	1913-2013	1913-2011
Newey-West bandwidth	21	20

Notes: Each column shows coefficients from a time-series regression of log commercial landing on current and past NAO and a 4th-order polynomial time trend. Serial correlation and heteroscedasticity robust Newey-West standard errors with optimal bandwidth. *** p<0.01, ** p<0.05, * p<0.1

Table N: Trend and lag selection tests on catch models in Gulf of Maine

	Number of trend terms				
	(1)	(2)	(3)	(4)	(5)
NAO_{t-1}					
Dickey-Fuller test	–	–	–	–	–
AIC	144.4	143.3	133.8	114.2	99.78
NAO_{t-2}					
Dickey-Fuller test	–	–	**	–	–
AIC	140.4	138.1	130.6	114.9	98.64
NAO_{t-3}					
Dickey-Fuller test	**	–	***	–	–
AIC	139.4	135.6	129.3	116.1	97.75
NAO_{t-4}					
Dickey-Fuller test	*	–	***	–	–
AIC	141.1	137.6	131.3	116.9	99.14
NAO_{t-5}					
Dickey-Fuller test	*	*	***	–	–
AIC	140.8	138.4	131.8	115	98.54
NAO_{t-6}					
Dickey-Fuller test	*	–	**	–	–
AIC	141.9	140.1	133.2	113.7	95.43
NAO_{t-7}					
Dickey-Fuller test	**	*	–	–	–
AIC	142.8	141.6	134.4	112.3	98.6
NAO_{t-8}					
Dickey-Fuller test	–	*	*	–	–
AIC	143	142.6	135.3	110.9	101.6
NAO_{t-9}					
Dickey-Fuller test	–	*	***	–	–
AIC	141.7	142.2	134.9	106.7	100.6
NAO_{t-10}					
Dickey-Fuller test	–	–	–	–	–
AIC	141.6	142.8	135.2	104.8	101.1
NAO_{t-11}					
Dickey-Fuller test	–	*	–	–	–
AIC	141.9	143.5	136.1	102.3	100.6
NAO_{t-12}					
Dickey-Fuller test	***	***	*	–	–
AIC	141.1	143	136	95.78	96.33
NAO_{t-13}					
Dickey-Fuller test	***	***	***	–	–
AIC	139.1	141.1	135.4	85.29	86.95
NAO_{t-14}					
Dickey-Fuller test	***	***	***	–	–
AIC	139.8	141.7	136.6	79.84	81.83
NAO_{t-15}					
Dickey-Fuller test	***	***	**	–	–
AIC	141.8	143.6	138.5	80.34	82.1
NAO_{t-16}					
Dickey-Fuller test	***	***	–	–	–
AIC	143.7	145.4	140.5	80.21	81.23
NAO_{t-17}					
Dickey-Fuller test	***	**	**	–	–
AIC	144.4	145.7	142.1	80.54	78.83
NAO_{t-18}					
Dickey-Fuller test	***	**	**	*	*
AIC	143.4	143.7	142.6	76.91	76.77
NAO_{t-19}					
Dickey-Fuller test	***	***	**	*	–
AIC	142.3	141.3	141.9	75.59	72.6
NAO_{t-20}					
Dickey-Fuller test	**	**	*	***	***
AIC	143.2	142.7	142.6	76.83	74.21

Notes: Each column shows statistics from a time-series regression of log commercial landing on current and past NAO. Order of polynomial time trend varies across columns. AIC statistic shown. Dickey-Fuller tests model residual against the presence of a unit root. *** $p<0.01$, ** $p<0.05$, * $p<0.1$, – $p>0.1$

Table O: Trend and lag selection tests on catch models in Georges Bank

	Number of trend terms				
	(1)	(2)	(3)	(4)	(5)
NAO_{t-1}					
Dickey-Fuller test	*	–	–	–	–
AIC	191	162	120.1	94.6	64.24
NAO_{t-2}					
Dickey-Fuller test	–	–	–	–	–
AIC	192.5	163.8	122.1	95.54	63.54
NAO_{t-3}					
Dickey-Fuller test	–	–	–	–	–
AIC	192.8	165.7	123.5	93.05	64.46
NAO_{t-4}					
Dickey-Fuller test	–	–	–	–	–
AIC	194.3	167.7	125.4	91.94	65.27
NAO_{t-5}					
Dickey-Fuller test	*	–	–	–	–
AIC	194.1	169.1	125.9	84.96	62.68
NAO_{t-6}					
Dickey-Fuller test	–	–	–	–	–
AIC	194.4	171	127.5	82.55	63.38
NAO_{t-7}					
Dickey-Fuller test	*	–	–	–	–
AIC	195.8	172.9	129.5	83.79	61.19
NAO_{t-8}					
Dickey-Fuller test	*	–	–	–	–
AIC	196.8	174.9	131.5	85.38	64.70
NAO_{t-9}					
Dickey-Fuller test	*	–	–	–	–
AIC	196.3	176.6	133.3	85.77	68.7
NAO_{t-10}					
Dickey-Fuller test	*	–	–	–	–
AIC	194.9	177.9	134.4	85.02	68.62
NAO_{t-11}					
Dickey-Fuller test	**	*	–	–	–
AIC	192.2	178.4	135.9	84.34	72
NAO_{t-12}					
Dickey-Fuller test	**	*	–	–	–
AIC	190.7	179.2	137.4	81.46	70.42
NAO_{t-13}					
Dickey-Fuller test	*	–	**	–	–
AIC	189.4	179.7	139.1	77.17	71.3
NAO_{t-14}					
Dickey-Fuller test	*	–	**	–	–
AIC	185.7	178.4	140.3	71.47	66.54
NAO_{t-15}					
Dickey-Fuller test	–	–	**	–	–
AIC	180.2	175.6	141.2	66.56	63.5
NAO_{t-16}					
Dickey-Fuller test	**	*	**	–	–
AIC	176.6	174.9	143.2	68.53	67.49
NAO_{t-17}					
Dickey-Fuller test	**	**	–	–	–
AIC	175.8	175.2	145	70.08	68.62
NAO_{t-18}					
Dickey-Fuller test	**	**	–	*	–
AIC	175.5	175.5	146.6	72	68.49
NAO_{t-19}					
Dickey-Fuller test	**	**	*	**	*
AIC	172.4	173.3	148.4	73.95	70.34
NAO_{t-20}					
Dickey-Fuller test	**	**	*	***	**
AIC	173.4	174.5	149.9	75.68	73.75

Notes: Each column shows statistics from a time-series regression of log commercial landing on current and past NAO. Order of polynomial time trend varies across columns. AIC statistic shown. Dickey-Fuller tests model residual against the presence of a unit root. *** p<0.01, ** p<0.05, * p<0.1, – p>0.1

Table P: Effects of SST on age-specific biomass in Gulf of Maine

	Dep. var. is log cohort-specific spring cod biomass											
	(1)	(2)	(3)	(4)	(5)	(6)	(7)	(8)	(9)	(10)	(11)	(12)
SST_t	1.305*	1.272**	0.343	0.285	0.00843	-0.209	0.00325	-0.0812	-0.401	-0.220	0.431	0.473
	[0.682]	[0.645]	[0.416]	[0.335]	[0.214]	[0.198]	[0.267]	[0.317]	[0.486]	[0.453]	[0.493]	[0.411]
SST_{t-1}	-0.240	-0.291	0.741	0.776*	0.342*	0.376	-0.0224	-0.0725	0.704**	0.824**	0.0747	0.0863
	[0.399]	[0.400]	[0.472]	[0.433]	[0.188]	[0.267]	[0.272]	[0.295]	[0.331]	[0.327]	[0.210]	[0.194]
SST_{t-2}			-1.125**	-1.229***	0.0188	0.109	-0.0253	-0.0491	0.241	0.359	0.487	0.531
			[0.480]	[0.469]	[0.263]	[0.236]	[0.219]	[0.239]	[0.255]	[0.263]	[0.393]	[0.479]
SST_{t-3}					-1.294*	-1.727***	-0.837***	-0.928***	-0.259	-0.0347	0.394*	0.466
					[0.716]	[0.571]	[0.271]	[0.359]	[0.471]	[0.612]	[0.205]	[0.356]
SST_{t-4}							-1.272***	-1.404***	-0.368	-0.123	-0.170	-0.0738
							[0.408]	[0.305]	[0.267]	[0.192]	[0.155]	[0.357]
SST_{t-5}									-0.808	-0.646	-0.0850	0.00983
									[0.594]	[0.662]	[0.101]	[0.264]
SST_{t-6}											-0.661	-0.665*
											[0.432]	[0.402]
$catch_{t-1}$		-0.0764*		-0.0819		-0.167***		-0.0545		0.0945**		0.0306
		[0.0396]		[0.0528]		[0.0307]		[0.0420]		[0.0418]		[0.0689]
adult $biomass_{t-age}$	0.151	0.146	0.223	0.252	0.287**	0.437***	0.0525	0.0791	0.164	0.156	-0.185	-0.144
	[0.156]	[0.151]	[0.357]	[0.374]	[0.144]	[0.139]	[0.113]	[0.0961]	[0.195]	[0.209]	[0.277]	[0.377]
ln[adult $biomass_{t-age}$]	-0.582	-0.486	-1.041	-1.106	-1.643*	-2.290***	-0.267	-0.427	-0.553	-0.441	1.094	0.943
	[0.750]	[0.780]	[1.752]	[1.813]	[0.878]	[0.886]	[0.485]	[0.424]	[0.887]	[0.859]	[1.529]	[1.889]
Observations	31	31	30	30	29	29	28	28	27	27	26	26
Sample period	1983-2013	1983-2013	1984-2013	1984-2013	1985-2013	1985-2013	1986-2013	1986-2013	1987-2013	1987-2013	1988-2013	1988-2013
Number of trends	3	3	3	3	3	3	3	3	3	3	3	3
Newey-West bandwidth	16	16	15	14	13	13	12	10	15	15	15	15

Notes: Each column shows coefficients from a time-series regression model of log cohort-specific spring cod biomass on current and past SST, adult biomass (ages 2 and up) from the spawning year of that cohort, and trend terms. Some models additionally include control for previous year catch. Coefficients shaded in gray capture birth-year SST effects. Serial correlation and heteroscedasticity robust Newey-West standard errors with optimal bandwidth. *** $p<0.01$, ** $p<0.05$, * $p<0.1$

Table Q: Effects of SST on age-specific biomass in Georges Bank

	Dep. var. is log cohort-specific spring cod biomass											
	(1)	(2)	(3)	(4)	(5)	(6)	(7)	(8)	(9)	(10)	(11)	(12)
	age=1	age=1	age=2	age=2	age=3	age=3	age=4	age=4	age=5	age=5	age=6	age=6
mean biomass (in kg)	0.12	0.12	0.79	0.79	2.41	2.41	2.76	2.76	2.68	2.68	1.29	1.29
SST_t	0.377**	0.325*	0.0636	0.0600	-0.0781	-0.0739	0.426***	0.442***	-0.0917	-0.0816	-0.532*	-0.536*
	[0.160]	[0.175]	[0.199]	[0.193]	[0.0721]	[0.0949]	[0.0882]	[0.107]	[0.0947]	[0.0956]	[0.302]	[0.299]
SST_{t-1}	-0.145	-0.0806	0.110	0.0888	-0.0688	-0.121	-0.252**	-0.232*	0.196	0.252	-0.0284	0.0479
	[0.155]	[0.183]	[0.126]	[0.154]	[0.105]	[0.132]	[0.117]	[0.119]	[0.129]	[0.172]	[0.221]	[0.286]
SST_{t-2}			-0.319	-0.361*	-0.00877	-0.0148	0.0693	0.0834	0.319***	0.355**	0.697***	0.700***
			[0.232]	[0.207]	[0.120]	[0.138]	[0.137]	[0.105]	[0.111]	[0.150]	[0.241]	[0.250]
SST_{t-3}					-0.344	-0.357	0.210**	0.217**	-0.214	-0.181	-0.225	-0.208
					[0.237]	[0.242]	[0.103]	[0.107]	[0.169]	[0.169]	[0.225]	[0.250]
SST_{t-4}							0.163	0.183	0.856***	0.891***	0.312*	0.414*
							[0.157]	[0.219]	[0.226]	[0.267]	[0.160]	[0.225]
SST_{t-5}									0.416	0.455	0.754***	0.772***
									[0.317]	[0.346]	[0.186]	[0.211]
SST_{t-6}											0.203	0.128
											[0.247]	[0.213]
$catch_{t-1}$		0.0444		-0.0218*		-0.0171		0.00544		0.0174		0.0477***
		[0.0322]		[0.0117]		[0.0208]		[0.0175]		[0.0127]		[0.00940]
adult $biomass_{t-age}$	-0.0407***	-0.0453***	-0.0488***	-0.0484***	-0.0393***	-0.0420***	-0.0176***	-0.0170**	-0.0542***	-0.0488***	-0.0327***	-0.0234*
	[0.0101]	[0.00858]	[0.00725]	[0.00899]	[0.00654]	[0.00997]	[0.00614]	[0.00765]	[0.00638]	[0.00580]	[0.01000]	[0.0121]
ln[adult $biomass_{t-age}$]	1.126***	1.004***	0.775***	0.862***	0.490***	0.600**	-0.242*	-0.269	0.665***	0.613***	0.0640	0.0134
	[0.417]	[0.370]	[0.158]	[0.195]	[0.190]	[0.294]	[0.142]	[0.167]	[0.136]	[0.127]	[0.270]	[0.263]
Observations	29	29	28	28	27	27	26	26	25	25	24	24
Sample period	1983-2011	1983-2011	1984-2011	1984-2011	1985-2011	1985-2011	1986-2011	1986-2011	1987-2011	1987-2011	1988-2011	1988-2011
Number of trends	3	3	3	3	3	3	3	3	3	3	3	3
Newey-West bandwidth	16	16	16	16	11	9	15	15	15	15	14	15

Notes: Each column shows coefficients from a time-series regression model of log cohort-specific spring cod biomass on current and past SST, adult biomass (ages 2 and up) from the spawning year of that cohort, and trend terms. Some models additionally include control for previous year catch. Coefficients shaded in gray capture birth-year SST effects. Serial correlation and heteroscedasticity robust Newey-West standard errors with optimal bandwidth. *** p<0.01, ** p<0.05, * p<0.1

Table R: Effects of NAO on fall-surveyed age-specific biomass in Gulf of Maine

	Dep. var. is log cohort-specific fall cod biomass											
	(1)	(2)	(3)	(4)	(5)	(6)	(7)	(8)	(9)	(10)	(11)	(12)
	age=1	age=1	age=2	age=2	age=3	age=3	age=4	age=4	age=5	age=5	age=6	age=6
mean biomass (in kg)	0.13	0.13	0.64	0.64	1.18	1.18	1.49	1.49	1.19	1.19	0.93	0.93
NAO_t	-0.0350	-0.0337	0.0533	0.0642*	0.0234	0.0390	-0.152***	-0.149***	0.0533	0.0773	-0.135	-0.171*
	[0.0544]	[0.0504]	[0.0466]	[0.0383]	[0.0288]	[0.0634]	[0.0439]	[0.0489]	[0.0520]	[0.214]	[0.0987]	[0.100]
NAO_{t-1}	-0.0430	-0.0374	0.0355	0.0406	0.0448	0.0832**	-0.0919	-0.0871	-0.147***	-0.139	-0.168***	-0.196***
	[0.144]	[0.144]	[0.110]	[0.109]	[0.0476]	[0.0388]	[0.0641]	[0.0604]	[0.0330]	[0.112]	[0.0532]	[0.0358]
NAO_{t-2}			-0.0931***	-0.0732***	-0.00789	0.0509	-0.0450	-0.0284	0.0250	0.0568	-0.0952	-0.163*
			[0.0163]	[0.0284]	[0.0309]	[0.0680]	[0.0383]	[0.0370]	[0.0276]	[0.200]	[0.0682]	[0.0862]
NAO_{t-3}					-0.243***	-0.186**	-0.196***	-0.181***	-0.0616	-0.0380	-0.128**	-0.188***
					[0.0329]	[0.0752]	[0.0321]	[0.0343]	[0.0381]	[0.0531]	[0.0510]	[0.0689]
NAO_{t-4}							-0.211***	-0.210***	0.0692**	0.0785	-0.143***	-0.144***
							[0.0218]	[0.0245]	[0.0350]	[0.0630]	[0.0351]	[0.0349]
NAO_{t-5}									-0.257***	-0.278**	-0.301***	-0.267***
									[0.0570]	[0.125]	[0.0700]	[0.0536]
NAO_{t-6}											-0.211***	-0.210***
											[0.0601]	[0.0618]
$catch_{t-1}$		-0.0572*		-0.0430		-0.135		-0.0341		-0.0717		0.135*
		[0.0330]		[0.0399]		[0.130]		[0.0286]		[0.338]		[0.0698]
adult $biomass_{t-age}$	0.0647	0.0640	0.126***	0.110***	-0.00548	-0.0576	0.105	0.0979	-0.0870**	-0.0821	0.0831***	0.0655
	[0.0742]	[0.0716]	[0.0398]	[0.0349]	[0.0669]	[0.0460]	[0.0723]	[0.0684]	[0.0350]	[0.138]	[0.0275]	[0.0475]
ln[adult $biomass_{t-age}$]	-0.0152	-0.00771	-1.462***	-1.299***	-0.351	0.132	-1.264**	-1.196**	0.103	-0.0136	-0.647**	-0.362
	[0.634]	[0.628]	[0.405]	[0.356]	[0.438]	[0.281]	[0.531]	[0.490]	[0.520]	[1.951]	[0.296]	[0.363]
Observations	41	41	40	40	39	39	38	38	37	37	36	36
Sample period	1971-2011	1971-2011	1972-2011	1972-2011	1973-2011	1973-2011	1974-2011	1974-2011	1975-2011	1975-2011	1976-2011	1976-2011
Number of trends	3	3	3	3	3	3	3	3	3	3	3	3
Newey-West bandwidth	17	17	17	17	17	17	17	17	17	17	16	16

Notes: Each column shows coefficients from a time-series regression model of log cohort-specific fall cod biomass on current and past NAO, adult biomass (ages 2 and up) from the spawning year of that cohort, and trend terms. Some models additionally include control for previous year catch. Coefficients shaded in gray capture birth-year NAO effects. Serial correlation and heteroscedasticity robust Newey-West standard errors with optimal bandwidth. *** $p<0.01$, ** $p<0.05$, * $p<0.1$

Table S: Effects of NAO on fall-surveyed age-specific biomass in Georges Bank

	Dep. var. is log cohort-specific fall cod biomass											
	(1)	(2)	(3)	(4)	(5)	(6)	(7)	(8)	(9)	(10)	(11)	(12)
	age=1	age=1	age=2	age=2	age=3	age=3	age=4	age=4	age=5	age=5	age=6	age=6
mean biomass (in kg)	0.270	0.270	0.630	0.630	1.040	1.040	0.790	0.790	0.380	0.380	0.460	0.460
NAO_t	0.0357	-0.0462	0.110*	0.107	0.0243	-0.0241	0.0452	0.0283	0.0244	0.0620	-0.0855	-0.168*
	[0.0484]	[0.0457]	[0.0579]	[0.0951]	[0.0756]	[0.0882]	[0.0709]	[0.0970]	[0.0270]	[0.114]	[0.0777]	[0.0908]
NAO_{t-1}	0.0531	0.0285	0.0229	0.0212	0.0983	0.0830	-0.136	-0.140	-0.0202	-0.0181	0.164***	0.0302
	[0.0958]	[0.0720]	[0.0502]	[0.0573]	[0.0676]	[0.0765]	[0.0926]	[0.0974]	[0.0248]	[0.0239]	[0.0473]	[0.0433]
NAO_{t-2}			-0.0398	-0.0412	-0.0236	-0.0537	0.115**	0.103***	-0.0308	-0.0147	-0.0976	-0.0763
			[0.0369]	[0.0262]	[0.0474]	[0.0529]	[0.0506]	[0.0385]	[0.0269]	[0.0422]	[0.0618]	[0.0884]
NAO_{t-3}					0.0145	-0.00687	-0.149***	-0.156***	-0.147***	-0.139***	0.0439	0.0261
					[0.0380]	[0.0498]	[0.0421]	[0.0341]	[0.0234]	[0.0281]	[0.0329]	[0.0439]
NAO_{t-4}							0.120*	0.126**	0.0737***	0.0664	-0.0184	0.0831*
							[0.0634]	[0.0605]	[0.0277]	[0.0434]	[0.0480]	[0.0485]
NAO_{t-5}									0.0180	0.00348	0.0825	0.196
									[0.0544]	[0.0465]	[0.0835]	[0.127]
NAO_{t-6}											-0.219*	-0.0619
											[0.117]	[0.0788]
$catch_{t-1}$		0.0534***		0.00239		0.0301		0.0120		-0.0174		0.128**
		[0.00971]		[0.0249]		[0.0304]		[0.0242]		[0.0405]		[0.0578]
adult $biomass_{t-age}$	-0.0709	-0.0445	0.0442	0.0424	0.0450	0.0548	-0.214**	-0.202***	-0.0208	-0.0192	-0.243***	-0.194
	[0.164]	[0.0656]	[0.0463]	[0.0509]	[0.166]	[0.166]	[0.0886]	[0.0782]	[0.0746]	[0.0860]	[0.0861]	[0.174]
ln[adult $biomass_{t-age}$]	-0.298	-0.525	-0.552**	-0.554	-0.466	-0.479	-0.342	-0.377	-0.582**	-0.657*	0.787**	1.150**
	[0.302]	[0.379]	[0.271]	[0.340]	[0.662]	[0.647]	[0.400]	[0.361]	[0.239]	[0.383]	[0.318]	[0.482]
Observations	33	33	32	32	31	31	30	30	29	29	28	28
Sample period	1979-2011	1979-2011	1980-2011	1980-2011	1981-2011	1981-2011	1982-2011	1982-2011	1983-2011	1983-2011	1984-2011	1984-2011
Number of trends	3	3	3	3	3	3	3	3	3	3	3	3
Newey-West bandwidth	16	16	16	16	16	16	16	16	16	16	14	10

Notes: Each column shows coefficients from a time-series regression model of log cohort-specific fall cod biomass on current and past NAO, adult biomass (ages 2 and up) from the spawning year of that cohort, and trend terms. Some models additionally include control for previous year catch. Coefficients shaded in gray capture birth-year NAO effects. Serial correlation and heteroscedasticity robust Newey-West standard errors with optimal bandwidth. *** p<0.01, ** p<0.05, * p<0.1
